# What do consumers understand about predispute arbitration agreements? an empirical investigation

**DOI:** 10.1371/journal.pone.0296179

**Published:** 2024-02-23

**Authors:** Roseanna Sommers

**Affiliations:** Michigan Law School, University of Michigan, Ann Arbor, Michigan, United States of America; Bryant University, UNITED STATES

## Abstract

The results of a survey of 1,071 adults in the United States reveal that most consumers do not pay attention to, let alone understand, arbitration clauses in their everyday lives. The vast majority of survey respondents (over 97%) report having opened an account with a company that requires disputes to be submitted to binding arbitration (e.g., Netflix, Hulu, Cash App, a phone or cable company), yet most are unaware that they have, in fact, agreed to mandatory arbitration (also known as “forced arbitration”). Indeed, over 99% of respondents who think they have never entered into an arbitration agreement likely have done so. Over 92% of respondents report that they have never based a decision to use a product or service on whether the terms and conditions contain an arbitration agreement. When prompted, they largely endorse the following reasons: they were unaware of the arbitration clause, they did not read the terms and conditions, and they thought they had no choice but to agree to mandatory arbitration. Moreover, many respondents presume that if a dispute arises, they will still be able to access the public courts, notwithstanding that they agreed to the terms and conditions. Consumers are largely unaware of opportunities to opt out of mandatory arbitration. They generally do not pay attention to or retain information about the steps required to opt out successfully (e.g., contacting the company within a specified time period). Generally, consumers are unaware that companies like Cash App and Venmo (mobile payment systems utilized by nearly 60% of respondents) allow customers to opt out of mandatory arbitration if they act within a limited time period. Among the minority of respondents (21%) who stated that they had been given an opportunity to opt out, vanishingly few could name any of the steps required to opt out successfully. When presented with a run-of-the-mill contract, of the type consumers routinely encounter, most respondents did not take notice of the arbitration clause. Less than 5% of respondents could recall that the contract they were shown had said anything at all about arbitration. Furthermore, most consumers misperceive the consequences of signing a predispute arbitration agreement. Most mistakenly believe that, after agreeing to terms and conditions mandating binding arbitration, they can still choose to settle their dispute in court, have a jury decide their case, join a class action, and appeal a decision made based on a legal error. For instance, less than 5% of respondents correctly reported that they could neither appeal an erroneous decision to another arbitrator (or set of arbitrators) nor start all over again in court. Less than 1% of respondents correctly understood the full significance of the arbitration agreement, as indicated by their responses to questions about whether they retained the rights to sue, have a jury decide their case, access the public courts, and appeal a decision based on a legal error. In summary, consumers are generally unaware of arbitration clauses, and they tend to hold mistaken beliefs about how arbitration agreements affect consumers’ procedural rights.

## Introduction

Arbitration clauses, also known as “mandatory arbitration” clauses or “forced arbitration” clauses, are contractual provisions agreed to in advance of any dispute or claim [1]. They require parties to submit any claims that may later arise to arbitration instead of taking them to court [2].

Arbitration clauses have proliferated in consumer contracts in recent years [3–5], and are “no longer the province of sophisticated participants” [6]. They are routinely presented to consumers within contracts of adhesion, which are standardized, preprinted form contracts offered to consumers on a take-it-or-leave-it basis [1].

Predispute arbitration agreements have been the subject of controversy. Critics charge that arbitration agreements “subject consumers to sharply unfair dispute resolution procedures” [7]. Writing in 2004, professors Linda Demaine and Deborah Hensler noted “heated[] debates” among legal commentators over the merits of a system in which consumer contracts “substitute binding arbitration for the public court system… in the course of their relationship with service or product providers” [6].

A crucial aspect of the controversy has concerned the degree to which consumers meaningfully consent to predispute arbitration agreements [3,4]. “Perhaps most central to the debate are concerns that consumers do not fully understand the terms of these agreements,” write Demaine and Hensler [6]. According to the National Consumer Law Center, “Most consumers do not focus on or understand the significance of waiving their future access to the public court system in the event that a dispute arises” [1].

This matters, some commentators have argued, because “[t]he legal regime supporting arbitration—and justifying the waiver of constitutionally protected procedural rights implicit in it—rests on the principle of consent” [2]. If arbitration agreements rest on tenuous consent, as many consumer advocates have claimed, it raises “serious questions” about “the assumptions underlying the law of arbitration” [2].

**Fig 1:**
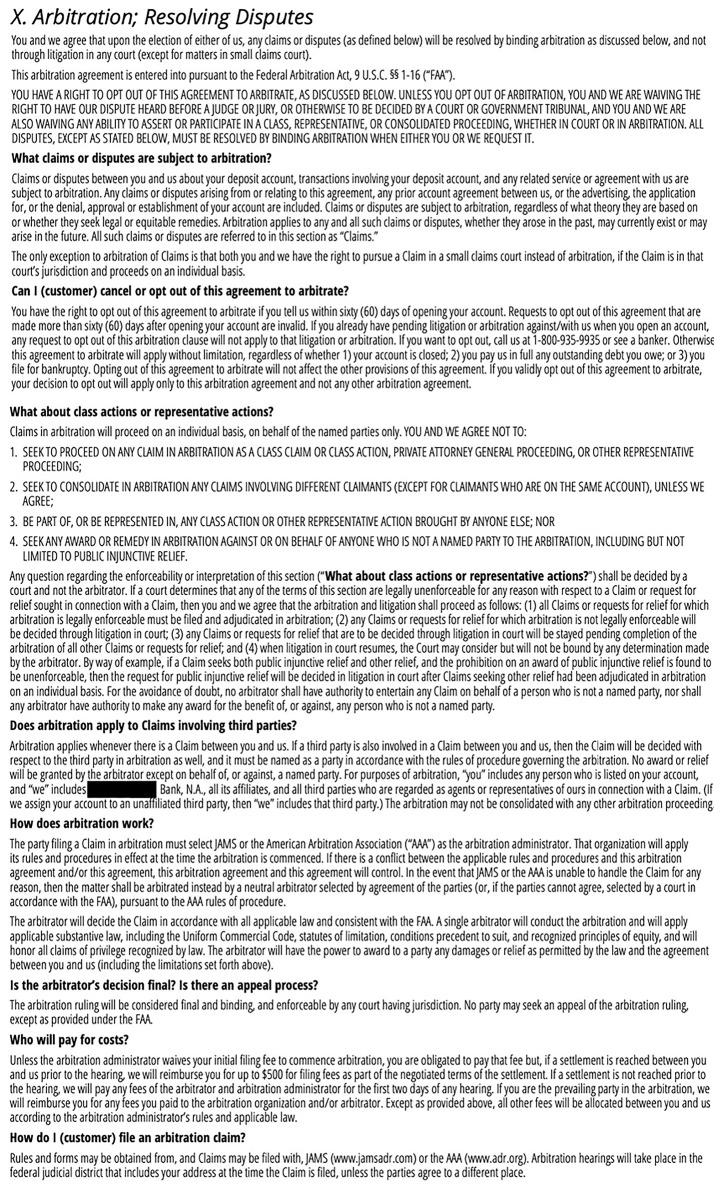
The arbitration agreement shown to survey respondents.

Are consumers being “unwittingly… stripped” [2] of important procedural rights? Some arbitration agreements contain provisions permitting consumers to opt out of or reject arbitration clauses if they take certain steps within a given time period. For example, Chase Bank allows consumers to opt out of mandatory arbitration if they contact the bank within sixty days of opening an account. See Fig 1 (“You have the right to opt out of this agreement to arbitrate if you tell us within sixty (60) days of opening your account. Requests to opt out of this agreement that are made more than sixty (60) days after opening your account are invalid…. If you want to opt out, call us at 1-800-935-9935 or see a banker.”).

It has been argued that such opt-out provisions give consumers choice and make consent meaningful [8]. According to this logic, the opportunity to opt out “means that the contract is not a take-it-or-leave-it contract of adhesion,” and thereby affords consumers choice over how disputes will be resolved [9]. Indeed, some courts have enforced arbitration clauses at least in part because an opt-out provision was present [9]. The Ninth Circuit, for example, has concluded that a “meaningful” opportunity to opt out of an arbitration requirement prevents a finding that the contract is procedurally unconscionable [8,9].

But do consumers actually attend to and understand opt-out provisions? The Consumer Financial Protection Bureau conducted a study in 2014 (published in 2015) that found that although 27.3% of credit card arbitration agreements contained opt-out provisions, very few consumers (0.3%) believed they had been given an opportunity to opt out of mandatory arbitration, and none of them had done so [10]. More generally, research from behavioral economics establishes that people tend to stick with default options, even when the transaction costs of opting out are minimal [11–13]. This suggests that consumers would seldom opt out of mandatory arbitration even if doing so were easy—and it is often not easy. For instance, Venmo customers who wish to opt out of mandatory arbitration must submit *by mail* a written notice that must be printed from Venmo’s website. The notice must be postmarked within 30 days of accepting the company’s User Agreement [14].

Consumer advocates, for their part, have argued that the “theory… that opt-out language ensures a voluntary choice is not borne out by reality” [8]. These commentators contend that “companies have been willing to adopt opt-out language as a strategy because they know very few potential class members read standard-form contracts, understand them, understand the differences between arbitration and litigation, are able to assess those differences, and have time to reject the default arbitration option by exercising any opt-out right” [8]. Data on consumer beliefs and behavior would help assess the claim that opt-out provisions make consumers’ consent to arbitration procedures more voluntary.

Prior empirical research suggests that consumers “are generally unaware” of arbitration agreements [10]. A prominent study from 2015 revealed a “profound lack of understanding” among consumers “about the existence and effect of arbitration clauses” [2]. After surveying 668 consumers about their own experiences with consumer contracts and showing survey respondents a typical credit card contract with an arbitration clause, law professor Jeff Sovern and colleagues concluded that “citizens are giving up these rights unknowingly, either because they do not realize they have entered into an arbitration agreement or because they do not understand the legal consequences of doing so” [2].

The present research seeks to update and expand upon this prior work. In particular, it seeks to replicate and extend Sovern and colleagues’ study examining the quality of consent that consumers typically provide to arbitration agreements.

In a survey conducted with a U.S. Census-matched sample of consumers, this research investigates five key questions:

1. When consumers reflect on their own contracting behavior, do they believe they have ever agreed to be bound by a mandatory predispute arbitration agreement?
2. Are consumers’ contracting decisions affected by the presence or absence of a mandatory arbitration clause? If so, in what ways? If not, why not?
3. Are consumers aware of provisions allowing them to opt out of arbitration?
4. What do consumers understand about the process of opting out of arbitration clauses? Do they take note of opt-out provisions that are presented to them?
5. What beliefs do consumers hold about the legal ramifications of entering into an agreement containing an arbitration clause, including the ability to have a dispute heard in court, the right to a jury trial, the possibility of joining a class action, and the ability to appeal an erroneous decision?

## Materials and methods

In June 2023, a sample of 1291 U.S.-based consumers was invited to take part in an academic survey. The study was approved by the Health Sciences and Behavioral Sciences Institutional Review Board at the University of Michigan. Participants provided written informed consent before beginning the survey.

Survey respondents were recruited from a nationwide survey panel curated by Centiment, a survey research firm [15]. The sample specifications targeted a general U.S. audience of 18+ years, with demographic balancing (+/- 3% of the U.S. Census) on gender; age group (18–34, 35–54, and 55+); U.S. Census region; and race/ethnicity. The specification further targeted a sample that was 50% above the national median household income of $70,000. The target size of the final sample was 1000 respondents; Centiment over-recruited to account for low-quality responses that would be excluded based on predetermined criteria.

This survey was preregistered at AsPredicted (https://aspredicted.org/TYL_QT2). The original survey materials and data are publicly available on the Open Science Framework (https://osf.io/c9vt6/).

In this study, consumers were shown a run-of-the-mill consumer contract—Chase Bank’s deposit account agreement—and asked to consider what, under the terms of the agreement, their options would be if various hypothetical situations arose. They were also asked to report their own prior experiences with predispute arbitration agreements. Chase Bank’s deposit account agreement and privacy notice was redacted to obscure that it was drafted by Chase. The stimuli presented to respondents is viewable at https://osf.io/a23gp.

### Procedure

At the beginning of the survey, respondents were asked to supply demographic information about their self-reported gender, age, race/ethnicity, level of educational attainment, household income, and legal experience. Next, they were asked whether they currently have an account with any of 16 services and products (e.g., a phone company, cable company, Netflix, Cash App), all of which were chosen because they require customers to agree to mandatory predispute arbitration as part of the standard terms and conditions.

Before being shown Chase Bank’s deposit account agreement, respondents were instructed: “On the page that follows, you will see a contract. We’d like you to read it the way you normally read contracts in your everyday life. Afterward, we will ask you some questions.”

Respondents were then shown the entirety of Chase Bank’s “Deposit Account Agreement and Privacy Notice.” This is a 28-page document consisting of 10 sections laid out in a table of contents. Section X is “Arbitration; Resolving Disputes” and spans less than two pages (Fig 1).

Public opinion polling from 2015 finds that consumers disfavor mandatory arbitration when it comes to resolving disputes between banks and their customers: a survey of 1,000 likely voters in the 2016 national election found that 75% supported the statement “Bank customers must have the right to take complaints to court, instead of being required to accept dispute arbitration by a third party chosen by the bank or lending institution” [16]. Thus, the deposit account agreement contains the kind of run-of-the-mill arbitration agreement that consumers tend to dislike [17].

The purpose of showing respondents a real consumer contract was to ensure that the manner in which terms were presented—including the use of boldface type, ALL-CAPS, and so on—was consistent with current practice.

After reviewing the contract, survey respondents were asked a series of recall and comprehension questions. The purpose of these questions was to determine how much respondents had absorbed and retained about the terms of the contract. The items were adapted from Sovern and colleagues’ 2015 survey, which measured responses to a credit card agreement.

Next, respondents were asked to consider a series of hypothetical scenarios. First, what would happen if they opened an account with the bank, and a security breach resulted in a costly event that the bank refused to remedy? Under the terms of the agreement they had seen earlier, would they be able to bring their dispute to court, have a jury hear the case, band together with other similarly situated bank customers in a class action, or appeal an arbitrator’s decision that was the result of a legal error? Suppose, hypothetically, they were physically injured by a security guard at a branch of the bank; under the agreement, would they have been able to sue the bank in court? Could they join other similarly situated individuals in a class action? The purpose of these questions was to determine the extent to which respondents understood the full significance and consequences of agreeing to a mandatory predispute arbitration agreement.

Respondents were also asked whether, under the agreement they’d been shown earlier, it would be possible to create an account with the bank *without* agreeing to mandatory arbitration. They were also asked to report what, if anything, they could recall about the steps the agreement said would need to be taken in order to opt out of the arbitration clause.

Respondents were additionally asked if they had ever entered into an arbitration agreement in their own lives, and if they had ever based a decision to obtain a consumer product or service on whether the terms and conditions contained an arbitration clause.

Respondents then indicated whether any of the 16 companies at which they had an account (determined by self-report) had permitted them to opt out of mandatory arbitration. They were also asked about specific companies they had reported giving business to (e.g., “Did your phone company ever give you a choice about whether you wanted to pre-commit to mandatory arbitration?”).

Next, they were asked whether their choice to open their account was affected by the presence of a mandatory arbitration clause. If they reported that their choice was unaffected, they were asked to rate their endorsement of four different statements: (*i*) they had been unaware of the arbitration clause; (*ii*) they did not read the terms and conditions when they made an account; (*iii*) they felt they had no choice but to accept all the terms and conditions; and (*iv*) they thought that they would still be able to resolve a dispute in court despite accepting the standard terms.

Finally, respondents were thanked and debriefed before they exited the survey.

## Results and discussion

### Sample characteristics

All told, 1291 respondents passed an attention check and were permitted to take the survey. Of these, 216 responses were excluded for writing gibberish in response to one or more open-ended questions, yielding a final sample of *n* = 1075, of which *n* = 944 answered all questions.

In the final sample, 47% were male, 53% were female, and less than 1% chose another designation. Respondents ranged in age from 18 to 88 years, with a median age of 46 years (*M*_age_ = 48 years, *SD*_age_ = 18 years). Most respondents (95%) reported being neither an attorney nor law student. Respondents’ self-reported racial/ethnic identification, educational attainment, and household income are reported in Tables 1.1–1.3.

**Table 1.1:** Respondents’ self-reported racial/ethnic identification.

Which racial or ethnic group in this list best describes you? You can select more than one. There are eight choices:	N	Percentage of sample
White (including Middle Eastern or Arab).	645	60%
Black/African-American	127	12%
Hispanic/Latino/a	114	11%
Asian	63	6%
American Indian/Alaska Native	2	<1%
Native Hawaiian/Other Pacific Islander	2	<1%
Other.	11	1.0%
Prefer not to answer	5	<1%
Chose one or more designation	102	10%
Total	1071	100%

**Table 1.2:** Respondents’ self-reported educational attainment.

Which is the highest level of education you have attained?	N	Percentage of sample
Did not graduate from high school	31	3%
High school graduate or GED	248	23%
Some college or post-secondary work	307	29%
College graduate	331	31%
Post-graduate work	156	15%
Total	1073	101%

**Table 1.3:** Respondents’ self-reported household income.

We will now ask about your total annual household income. There are six choices:	N	Percentage of sample
Less than $24,000	160	15%
At least $24,000 but less than $50,999	258	24%
At least $51,000 but less than $69,999	149	14%
At least $70,000 but less than $143,999	377	35%
At least $144,000	111	10%
Prefer not to answer	15	1%
Total	1070	100%

As described earlier, respondents were presented with a list of 16 common products and services that, by default, require consumers to accept mandatory arbitration as a condition of creating an account (Table 1.4). While some of these arbitration agreements provide consumers with the opportunity to opt out of binding arbitration, it is unlikely that any respondents availed themselves of these opportunities (see “Awareness of Opt-Out Provisions” Section). Thus, this research assumes that a person who reports holding an account with one of these firms has agreed to be bound by a mandatory predispute arbitration agreement.

**Table 1.4:** What consumer products do respondents use?

Firm	N	Percentage
Phone company^a^	905	84%
Netflix	710	66%
Cash App	532	49%
Hulu	469	44%
Venmo	396	37%
Cable company^b^	383	36%
Apple Pay	323	30%
Zelle	320	30%
Wayfair	294	27%
Chime	206	19%
Intuit	197	18%
Afterpay	141	13%
Coinbase	137	13%
Klarna	113	11%
Checkr	82	8%
Tinder	70	7%

^a^ The item’s exact wording was: “I have an account with a cell phone company such as Verizon Wireless, AT&T, T-Mobile, or Sprint, and I am the primary person on the account (as opposed to being an authorized user on somebody else’s account).”

^b^ The item’s exact wording was: “I have an account with a cable company and am the primary person on the account (as opposed to being an authorized user on somebody else’s account.”

### Attention and recall

This study seeks to assess the quality of consent that consumers give to predispute binding arbitration agreements. A preliminary question is how closely consumers attend to arbitration clauses in the first place.

A page timer was used to observe how long respondents spent on the webpage displaying the Chase contract before moving on to the rest of the survey. The timer data reveal that the average amount of time respondents spent on the page displaying the contract was 37.21 seconds (*SD* = 35.21*s*)—far short of the time needed for the average adult to read a 28-page contracts [2].

(As pre-registered, outliers were identified based on the median absolute deviation (MAD) and were excluded from analysis of the timer data [18]. When outliers are included, the mean time respondents spent on the webpage displaying the contract was 2 minutes and 22 seconds (*M* = 141.97*s*, *SD* = 440.36*s*); following Sovern and colleagues’ assumption of a reading speed of 300 words per minute, this suggests that the average number of words read by a survey respondent was 710, which is far short of the 1,500+ words in the arbitration clause (let alone the entire contract). The median time respondents spent on the webpage displaying the contract was 32.85 seconds; the median was 23.5 seconds when outliers are excluded. The mean absolute deviation (MAD) indicator was 37.62 seconds.)

The finding that respondents tend to spend very little time reading the contract is consistent with prior empirical work examining how consumers interact with contracts of adhesion [2,19–27]. It is possible, of course, that participants in the present study were particularly likely to rush through the contract presented in the survey, and that consumers in more naturalistic settings would spend more time reading and absorbing contractual language. But real-world data suggest that vanishingly few consumers in everyday settings read contracts in their entirety [21,26].

What did consumers recall about the contract they had just seen? Following the procedure used by Sovern and colleagues, respondents were instructed: “The contract you just saw said many things. We would like to know what you remember. Please put down a word or phrase for five items you recall…. If you don’t remember five items, please mention as many or as few as you do remember” [2].

Respondents filled in up to five open-ended text boxes. In total, 1075 respondents produced 3188 entries, of which 1228 were unique words or phrases. The most frequently named item was “none” (*n* = 145), followed by “annual fee” (*n* = 122), a term that did not appear in the contract they were shown. The next most frequently named items were “fee” or “fees” (*n* = 117); “deposit” (*n* = 108); “account” (*n* = 71); “ATM” (*n* = 49); and “agreement” (*n* = 45).

The key question for the purposes of this study is whether participants attended to the arbitration clause contained within Section X of the agreement. Results indicate that 27 respondents (2.5% of the total sample) explicitly mentioned “arbitration.” Another 22 respondents (2.0%) mentioned items that either appeared in the arbitration section of the contract (e.g., “dispute”; “binding”; “waived”)—although they appeared elsewhere in the contract as well—or arguably referenced topics covered in the arbitration section (e.g., “filing a lawsuit”; “liability limits”; “cannot sue”).

All told, references to the arbitration section of the contract made up 1.4% of the total mentions. Thus, consistent with prior empirical research findings, few respondents focused on dispute-resolution terms, or indeed read the fine print at all, when encountering a consumer contract.

One might wonder whether consumers who agree to arbitration without reading the terms nonetheless understand—or at least would not be surprised by—the consequences of choosing to agree to mandatory arbitration. That is, even if they forgo the opportunity to read the terms, they may still appreciate the possible ramifications of the choice to waive their right to access the public courts. The next section investigates what beliefs consumers hold about their rights after signing an arbitration agreement.

### What rights do respondents believe they retain after signing an arbitration agreement?

Do consumers understand the significance of waiving their future access to the public court system? Consumer advocates have raised concerns about several features of mandatory arbitration: the secrecy and lack of transparency (which hinder the public’s ability to scrutinize decisions for bias and other problems), limitations on discovery (the scope of which is left to the discretion of the arbitrator), and cumbersome provisions requiring people to arbitrate their claims in inconvenient geographic locations [3,17,28,29]. Furthermore, a 2012 survey commissioned by Pew found that “consumers overwhelmingly find the majority of components of arbitration unacceptable,” and that this holds true for respondents of “every major political affiliation” [17]. The present study will focus on four key procedural rights that are typically waived when consumers agree to mandatory arbitration.

1. *Right to access the public courts*. In a mandatory predispute arbitration agreement, parties agree by contract to waive their rights to resolve future disputes in a public courtroom, even if one of the parties wishes to proceed in court [30].
2. *Right to a jury trial*. Arbitration requires consumers to waive their right to a jury trial. Courts have determined that arbitration is not unconstitutional because it does not involve state action [31].
3. *Right to participate in a class action*. Most arbitration agreements come with class action waivers, which prevent consumers from banding together to bring claims on a class-wide basis [3,32]. The result, according to law professor Judith Resnik, has been the “mass production of arbitration clauses without a mass of arbitrations” [33].
4. *Limited appeal*. In binding arbitration, arbitrators are empowered to issue a final, binding ruling that is subject to “very limited judicial review” [1,10]. Unfortunately, arbitration decisions may fail to comport with applicable law, but “[b]ecause the [Federal Arbitration Act] provides the consumer with a sharply circumscribed ability to appeal the decision maker’s erroneous interpretation of the law, arbitrators may effectively ignore state or federal consumer protection statutes and judicial precedent” [31]. There is no right to appeal the arbitrator’s decision and no ability to start over in court, even when the decision is the product of an erroneous application of the law [1]. “The lack of an appeals process means that even grossly erroneous applications of the law are generally binding,” according to the National Consumer Law Center [31].

### Identity theft scenario

First, the survey probed consumers’ beliefs about how mandatory arbitration would affect a dispute with the bank over remedying costly identity theft. This was Sovern and colleagues’ scenario about a dispute with a credit card company, adapted to be about a deposit account agreement with a bank [2]. Respondents were instructed:

You just read contractual language about opening an account with a bank. Imagine that you decide to open an account with the bank, accepting the terms and conditions you just saw. Imagine that a few years after you opened your account, you learned about a data breach that affected your account. Your personal information was exposed, which put you at risk of identity theft. As a result, you changed your passwords, added a security alert to your credit reports, and put a security freeze on your credit file. You also began to monitor all your accounts for suspicious activity. Unfortunately, you became the victim of identity theft. All told, the data breach ended up costing you thousands of dollars. Imagine that the bank disputes that the data breach caused these problems for you. The dispute is too large to be decided by a small claims court.

Respondents were then asked a series of questions, adapted from Sovern and colleagues’ survey, about what their rights would be in such a scenario:

- *Day in court*. Respondents were asked, “Under the terms of the contract you just saw, if the amount of the dispute was large enough, would you have the right to have a court decide the dispute even if the bank did not want a court to decide the dispute?”
- *Right to a jury trial*. “Under the terms of the contract you just saw, would you have a right to a jury trial?”
- *Class action*. “Suppose that you and many other customers, who also suffered identity theft resulting from the same data breach at the bank, all had the same type of dispute with the bank. Under the terms of the contract you just saw, could you be included with the other customers in a single lawsuit (that is, a class action lawsuit) against the bank?”

Each question was accompanied by three answer options: “Yes,” “No,” and “I don’t know.” Tables 2.1–2.3 report respondents’ answers.

**Table 2.1.** 

Would you have the right to have a court decide the dispute even if the bank did not want a court to decide the dispute?	N	Percentage of sample [95% confidence interval]
Yes	531	**56%** [53%, 59%]
No	151	**16%** [14%, 18%]
I don’t know	271	**28%** [26%, 31%]
Total	953	100%

**Table 2.2.** 

Under the terms of the contract you just saw, would you have a right to a jury trial?	N	Percentage of sample [95% confidence interval]
Yes	451	**48%** [44%, 51%]
No	205	**22%** [19%, 24%]
I don’t know	293	**31%** [28%, 34%]
Total	949	101%

**Table 2.3.** 

Under the terms of the contract you just saw, could you be included with the other customers in a single lawsuit (that is, a class action lawsuit) against the bank?	N	Percentage of sample [95% confidence interval]
Yes	573	**60%** [57%, 63%]
No	137	**14%** [12%, 17%]
I don’t know	239	**25%** [23%, 28%]
Total	949	99%

As Tables 2.1–2.3 show, most respondents wrongly believe they retain the right to sue in court, to have a jury decide the dispute, and to join a class action—or are unsure. Only a minority of respondents correctly ascertain that these rights have been forfeited under the terms of the contract.

Respondents were asked about a fourth procedural right: the right to an appeal. They were asked to imagine the agreement contained a “properly worded clause” mandating that “disputes could be resolved only in arbitration, and the arbitrator’s decision is final.” They were informed:

When a contract states that all disputes will be settled through arbitration, this is called ‘mandatory arbitration.’ It means that people who agree to the contractual language are required to resolve any disputes through privately appointed individuals (arbitrators), rather than through the court system. Thus, even if a customer wants to have the dispute settled in court, they are required to go to arbitration instead.

Next, respondents were instructed to imagine, once again, that the bank’s data breach had caused them to lose $5,000, but the bank disputed this. This time, respondents were instructed to imagine they had submitted the claim to arbitration and that the arbitrator had ruled for the bank as a result of the arbitrator’s mistake: “Assume that the arbitrator made a mistake about the law that caused them to rule against you. The mistake was not intentional. Assume that the arbitrator otherwise conducted the arbitration properly.”

They were instructed to assess the options available to them in this situation and to indicate if they agreed, disagreed, or neither agreed nor disagreed with two statements: (*i*) “I could appeal the arbitrator’s erroneous decision to another arbitrator or set of arbitrators”; and (*ii*) “I could set aside the arbitrator’s erroneous decision and start all over again in court.” Tables 2.4 and 2.5 show the results.

**Table 2.4.** 

I could appeal the arbitrator’s erroneous decision to another arbitrator or set of arbitrators.	N	Percentage of sample [95% confidence interval]
Agree	583	**62%** [58%, 65%]
Neither agree nor disagree	293	**31%** [28%, 34%]
Disagree	70	**7%** [6%, 9%]
Total	946	100%

**Table 2.5.** 

I could set aside the arbitrator’s erroneous decision and start all over again in court	N	Percentage of sample [95% confidence interval]
Agree	327	**35%** [32%, 38%]
Neither agree nor disagree	420	**44%** [41%, 48%]
Disagree	199	**21%** [19%, 24%]
Total	946	100%

Results indicate that only 7% of respondents correctly noted that they could not appeal to another arbitrator and only 21% correctly reported that they could not start over again in court. In total, only 4.9% of respondents (*n* = 46) correctly reported that they could neither appeal the decision to another arbitrator (or set of arbitrators) nor start over again in court.

Out of all 946 respondents who answered all five of the preceding questions, only 5 correctly reported that they did not retain the rights to sue in court, to a jury trial, to join a class action, to appeal an erroneous decision to another arbitrator or to start over again in court. Thus, only 0.5% of respondents correctly understood the full significance that the arbitration agreement carried for their procedural rights.

### Illegal detention scenario

Next, participants were asked to imagine a new hypothetical scenario involving a dispute with the bank: “Now we’d like you to imagine a different scenario involving the bank where you opened an account. Suppose that you experienced an upsetting incident at the bank in which a security guard wrongfully detained you—that is, the security guard physically prevented you from leaving the building. The guard did not have a legitimate reason to detain you. Furthermore, in the process of restraining you, the guard physically injured you.”

They were asked two questions about what their rights would be, having signed the agreement presented earlier, in such a scenario: (1) “Under the terms of the contract you saw, would you have a right to sue the bank in court?”; and (2) “Suppose that you and several other customers were all wrongfully detained by security guards at various branches of the bank. Under the terms of the contract you saw, could you be included with the other customers in a single lawsuit (that is, a class action lawsuit) against the bank?” The answer choices were “Yes,” “No,” and “I don’t know.” Tables 3.1 and 3.2 display the results.

**Table 3.1.** 

Under the terms of the contract you saw, would you have a right to sue the bank in court?	N	Percentage of sample [95% confidence interval]
Yes	546	**58%** [55%, 61%]
No	190	**20%** [18%, 23%]
I don’t know	209	**22%** [20%, 25%]
Total	945	100%

**Table 3.2.** 

Under the terms of the contract you saw, could you be included with the other customers in a single lawsuit (that is, a class action lawsuit) against the bank?	N	Percentage of sample [95% confidence interval]
Yes	530	**56%** [53%, 59%]
No	174	**18%** [16%, 21%]
I don’t know	241	**26%** [23%, 28%]
Total	945	100%

As Tables 3.1 and 3.2 show, respondents generally believe they retained the rights to sue in court and join a class action in the wrongful detention scenario. Yet prior case law suggests that they may be mistaken [34].

All told, of the 945 respondents who answered the two detention questions in addition to the five questions about the identity theft, only 2 answered all seven questions correctly. Thus, only 0.2% of respondents seemed to understand the significance of mandatory arbitration, including its consequences for their rights to sue in court, to have a jury decide the dispute, to join a class action, and to appeal an erroneous decision to another arbitrator or to start over again in court.

### How much experience do respondents have with arbitration clauses?

After responding to the hypothetical scenarios about the bank, respondents were asked about their personal experience with arbitration clauses: “We will now ask you some general questions about your own understanding and personal preferences about consumer contracts.” They were then asked if they personally had ever entered into a mandatory predispute arbitration agreement (Table 4.1). Sovern and colleagues used a similar instruction and measure in their 2015 survey [2].

**Table 4.1.** 

Have you ever entered into a consumer contract with a company that said you must arbitrate any disputes (and therefore cannot sue the company)?	N	Percentage of sample [95% confidence interval]
Yes	157	**17%** [14%, 19%]
No	455	**48%** [45%, 51%]
Unsure	333	**35%** [32%, 38%]
Total	945	100%

Most respondents were either unsure whether they had (35%), or asserted they had not (48%), agreed to be bound by an arbitration clause. At the same time, nearly all respondents (*n* = 1046, 97%) reported having one or more accounts with a firm that requires consumers to agree to mandatory arbitration clause as part of the standard terms and conditions. In other words, of the 48% of respondents (*n* = 455) who say they have never entered into an arbitration agreement, 99% (*n* = 451) self-report having an account with at least one company. This result underscores how little awareness consumers have about mandatory predispute arbitration agreements that they have putatively consented to.

### Does arbitration affect consumers’ contracting decisions ex ante?

Next, consumers were asked if they had ever decided to use a product or service based on whether the terms and conditions contain an arbitration clause (Table 5.1).

**Table 5.1.** 

Have you ever decided to use a product or service based on whether the terms and conditions contain an arbitration clause?	N	Percentage of sample [95% confidence interval]
Yes	67	**7%** [6%, 9%]
No	878	**93%** [91%, 94%]
Total	945	100%

Results indicate that the vast majority of consumers (93%) do not factor in mandatory arbitration when deciding to sign up for a product or service such as Netflix, Cash App, or a phone plan. This comports with the CFPB’s study, which found that for credit card users, “dispute resolution plays little to no role in choosing the credit card they use most often” [10]. The present study expands upon this inquiry by investigating whether dispute resolution procedures ever play a role, in any contracting decisions consumers make [2].

Respondents who answered that they have previously decided to use a product or service based on whether the terms and conditions contained an arbitration clause (*n* = 67) were asked to elaborate: “[P]lease describe a time when you decided to use a product or service based on whether the terms and conditions contained an arbitration clause. Please provide your reasoning.”

Many respondents failed to answer the question meaningfully, offering responses such as, “I really do not know” or “I’m not sure if it contained such a clause.” Respondents who gave responsive answers wrote statements such as: “I try to avoid contracts that demand arbitration, but it is not always a practical option.”; “Needed the loan.”; “It was the only way to obtain the service. If had not signed, would not have had the service.”; and “I believe it was for cable television.”

Later in the survey, respondents were asked to recall whether their decision to sign up for specific services was affected by the presence of an arbitration clause: “Think back on your choice to make an account with [Netflix]. Was your choice to make an account with [Netflix] affected by whether [Netflix]’s terms and conditions contained an arbitration clause?”

The bracketed language was replaced by one of the goods or services the respondent had previously reported using.

Respondents were asked only about products or services they reported using. In order to keep the survey to a reasonable length, respondents who reported holding accounts with multiple companies were asked about a maximum of three firms.

Results show that the overwhelming majority of respondents do not consider arbitration when deciding whether to make an account (Table 5.2).

**Table 5.2:** Was your choice to make an account with [firm] affected by whether5 [firm’s] terms and conditions contained an arbitration clause?

Firm	Yes		No		
	Estimate	CI	Estimate	CI	N
Phone company	**3%**	[2%, 5%]	**97%**	[95%, 98%]	510
Netflix	**2%**	[1%, 4%]	**98%**	[96%, 99%]	375
Cash App	**5%**	[3%, 8%]	**95%**	[92%, 97%]	270
Hulu	**2%**	[1%, 5%]	**98%**	[95%, 99%]	225
Cable company	**7%**	[4%, 11%]	**93%**	[89%, 96%]	214
Venmo	**0%**	[0%, 2%]	**100%**	[98%, 100%]	179
Apple Pay	**4%**	[2%, 8%]	**96%**	[92%, 98%]	155
Zelle	**4%**	[2%, 8%]	**96%**	[92%, 98%]	138
Wayfair	**3%**	[1%, 7%]	**97%**	[93%, 99%]	136
Intuit	**3%**	[1%, 9%]	**97%**	[91%, 99%]	95
Chime	**5%**	[2%, 12%]	**95%**	[88%, 98%]	78
Coinbase	**9%**	[4%, 20%]	**91%**	[80%, 96%]	53
Afterpay	**4%**	[1%, 15%]	**96%**	[85%, 99%]	46
Checkr	**5%**	[1%, 15%]	**95%**	[85%, 99%]	44
Klarna	**7%**	[3%, 20%]	**92%**	[80%, 97%]	40
Tinder	**0%**	[0%, 13%]	**100%**	[87%, 100%]	25

Respondents who said their choice was unaffected by the presence of an arbitration clause were presented with four statements and asked to choose “Agree”; “Disagree”; or “Neither Agree Nor Disagree”:

1. I was not aware of the company’s arbitration clause.
2. I did not read the company’s terms and conditions when I made an account.
3. I felt I had no choice but to accept all the company’s terms and conditions.
4. I thought that if something bad happened, I would still be able to sue the company in court.

Respondents could endorse or reject multiple statements; the four rationales were not mutually exclusive.

As Table 5.3 shows, most participants who say the presence of an arbitration clause did not affect their decision to create an account endorsed as reasons: (*i*) unawareness of the clause and (*ii*) not reading the terms and conditions. Furthermore, sizable percentages of respondents say that (*iii*) they felt they had no choice but to accept the terms and (*iv*) they held the (mistaken) belief that they would still be able to sue in court if a problem arose. This latter finding comports with the CFPB’s prior research showing that consumers’ beliefs about their dispute resolution rights “bear little to no relation” to the dispute resolution provisions of the contracts they sign [10].

**Table 5.3:** Percentage of participants selecting “Agree”.

	Not Aware of Clause		Did Not Read the Terms		No Choice But to Accept		Still Able to Sue in Court		
	Est.	CI	Est.	CI	Est.	CI	Est.	CI	N
Phone company	**62%**	[57%, 67%]	**51%**	[46%, 56%]	**52%**	[47%, 57%]	**36%**	[32%, 41%]	409
Netflix	**62%**	[57%, 67%]	**55%**	[49%, 60%]	**49%**	[43%, 54%]	**32%**	[27%, 37%]	311
Cash App	**56%**	[49%, 62%]	**45%**	[38%, 51%]	**43%**	[37%, 50%]	**36%**	[30%, 43%]	222
Hulu	**64%**	[57%, 71%]	**55%**	[48%, 62%]	**46%**	[39%, 53%]	**35%**	[29%, 42%]	195
Cable company	**64%**	[57%, 70%]	**54%**	[47%, 61%]	**54%**	[46%, 61%]	**42%**	[35%, 49%]	185
Venmo	**61%**	[53%, 69%]	**59%**	[51%, 67%]	**50%**	[42%, 58%]	**40%**	[32%, 48%]	149
Apple Pay	**62%**	[53%, 70%]	**54%**	[45%, 63%]	**58%**	[49%, 66%]	**39%**	[31%, 47%]	124
Wayfair	**72%**	[64%, 80%]	**66%**	[57%, 73%]	**49%**	[40%, 58%]	**44%**	[35%, 53%]	119
Zelle	**70%**	[61%, 78%]	**65%**	[56%, 73%]	**60%**	[51%, 69%]	**41%**	[32%, 50%]	111
Intuit	**56%**	[45%, 66%]	**51%**	[41%, 62%]	**46%**	[36%, 57%]	**28%**	[19%, 39%]	82
Chime	**38%**	[27%, 51%]	**36%**	[25%, 50%]	**44%**	[31%, 57%]	**40%**	[28%, 53%]	55
Coinbase	**67%**	[51%, 79%]	**56%**	[41%, 71%]	**69%**	[54%, 81%]	**28%**	[17%, 44%]	39
Afterpay	**47%**	[31%, 63%]	**56%**	[39%, 71%]	**50%**	[34%, 66%]	**32%**	[19%, 49%]	34
Checkr	**64%**	[47%, 78%]	**58%**	[41%, 73%]	**61%**	[44%, 75%]	**55%**	[38%, 70%]	33
Klarna	**59%**	[42%, 74%]	**66%**	[48%, 80%]	**53%**	[36%, 69%]	**44%**	[28%, 61%]	32
Tinder	**73%**	[52%, 87%]	**59%**	[39%, 77%]	**50%**	[31%, 69%]	**59%**	[39%, 77%]	22

### Awareness of opt-out provisions

It has been contended that consumers give meaningful consent to predispute arbitration when they are presented with the opportunity to opt out of arbitration clauses. But are consumers aware that they can opt out of mandatory arbitration? Do they understand what steps they must take in order to opt out successfully?

This question was explored, first, in the context of the deposit account agreement respondents were shown during the study. The Chase contract stated that customers are permitted to opt out of mandatory arbitration by calling the bank at a phone number provided or seeing a banker, so long as they did so within sixty days of opening the account (see Fig 1 “Can I (customer) cancel or opt out of this agreement to arbitrate?”). The survey asked respondents to recall whether opting out of the arbitration agreement was possible, and, if so, what steps would need to be taken.

After reading the Chase contract, respondents were asked whether the agreement allowed customers to opt out of mandatory arbitration:

[W]e’d like you to imagine that you decide to open an account with the bank, accepting the terms and conditions you saw earlier. The terms and conditions said that if you and the bank had a dispute, you couldn’t sue them in court. Instead, disputes must be resolved only in arbitration. Suppose that you objected to the idea of mandatory arbitration. Suppose you wanted to opt out of the part of the terms and conditions that states you must resolve all disputes in arbitration. According to the contractual language you read earlier, are you allowed to create an account with the bank, WITHOUT accepting the part of the agreement that says all disputes will be resolved only in arbitration?

Respondents’ beliefs about the availability of the opportunity to opt out of mandatory arbitration are reported in Table 6.1.

**Table 6.1:** 

According to the contractual language you read earlier, are you allowed to create an account with the bank, WITHOUT accepting the part of the agreement that says all disputes will be resolved only in arbitration?	N	Percentage of sample [95% confidence interval]
Yes	166	**18%** [15%, 20%]
No	467	**49%** [46%, 53%]
I don’t know	311	**33%** [30%, 36%]
Total	944	100%

Most respondents thought that customers had no choice but to agree to the predispute arbitration clause. Only 18% of respondents correctly reported that it was possible to create an account without agreeing to mandatory arbitration. Nearly half of participants (49%) mistakenly believed that opting out of mandatory arbitration was not possible, while another 33% were unsure.

Next, respondents were informed that the contract did, indeed, allow consumers to opt out of the arbitration clause. Respondents were asked to try to recall any of the steps that the contract said would be required in order to opt out: “The terms and conditions you saw earlier stated that users have the right to opt out of agreeing to resolve all disputes through arbitration if they take certain steps. Do you remember what any of those steps were? Please list them below. If you do not remember any of the steps, please write ‘I do not remember.’”

Results indicate that most respondents (*n* = 814, 86%) report that they did not remember. Among those who offered an answer, several (*n* = 42, 4.5%) gave nonsensical responses (e.g., “No,” “Agreement on a good deal,” “I think they should take the appropriate steps needed to win the suit.”).

Only 14 respondents (1.5%) gave answers that were broadly correct, such as “contact the company.” An additional 15 respondents (1.6%) gave answers that were arguably correct, meaning that although they did not accurately report the steps the contract laid out, they described actions that could plausibly result in a successful opt out (e.g., “File formal dispute within 30 days”; “I do not remember all of them but it starts with putting it in writing and submitting to a dept of the bank.”). Only 3 respondents (0.3%) mentioned the time limit of sixty days from the date the account was opened.

Thus, few respondents (less than 20%) believe opting out is possible and almost none (less than a third of one percent) understand that opting out would need to be done within sixty days of creating an account. The sixty-day window is especially significant because, in practice, it often means that consumers must opt out before any wrongdoing has occurred or been discovered.

The second way in which awareness of opt-out provisions was investigated involved asking respondents to reflect on their own accounts and whether they had been given the chance to opt out of arbitration when they initially signed up.

Respondents were asked: “Earlier, you stated that you have an account with the following companies: [List populated based on participants’ responses to earlier questions]. Did any of them allow you to opt out of the portion of the terms and services that said that disputes would be resolved through arbitration?” (Table 6.2)

**Table 6.2:** 

Did any of [Netflix/Hulu/Venmo/etc.] allow you to opt out of the portion of the terms and services that said that disputes would be resolved through arbitration?	N	Percentage of sample [95% confidence interval]
Yes	197	**21%** [18%, 24%]
No	284	**30%** [27%, 33%]
I don’t know	462	**49%** [46%, 52%]
Total	943	100%

If respondents answered “yes,” they were asked to “list all the accounts that allowed you to opt out below, and describe what, if anything, you remember about the steps you would have needed to take to opt out of the standard arbitration clause.” Only 2 respondents (0.21%) listed a firm that includes an opt-out provision in its arbitration agreement and described an action that could be taken to opt out. One respondent wrote, “venmo you would have to declare your intention in writing.” While this response is correct, it is unclear from the respondent’s answer whether the respondent was aware that Venmo requires users not only to declare their intention in writing, but also to submit their written notice to Venmo by mail within thirty days of accepting the user agreement [14]. (The other respondent wrote, “Cash app, request opt out,” which is correct but incomplete. Cash App’s user agreement states that consumers “must send us an opt-out notice (the “Opt Out”) within thirty (30) days after you create a Cash App Account,” which must be sent by mail [35]).

Next, respondents were provided additional information about (up to three) specific accounts they personally have a relationship with. They were told: “You indicated that you have an account with [Venmo]. [Venmo’s] standard terms and conditions say that all disputes will be settled through arbitration. This is called ‘mandatory arbitration.’ It means that even if a [Venmo] user wants to have the dispute settled in court, they are required to go to arbitration instead.”

Respondents were then asked, “Did [Venmo] ever give you a choice about whether you wanted to pre-commit to mandatory arbitration?” Table 6.3 reports the proportion of respondents endorsing each of three answer options, for each firm.

**Table 6.3:** Were you given a choice to opt out?

	Yes, I was given a choice to opt out of the standard arbitration language.		No, I was not given a choice.		I don’t remember if I was given a choice to opt out.		
	Est.	CI	Est.	CI	Est.	CI	N
Phone company	**15%**	[12%, 18%]	**33%**	[29%, 37%]	**52%**	[48%, 57%]	510
Netflix	**13%**	[10%, 17%]	**34%**	[29%, 39%]	**53%**	[48%, 58%]	373
Cash App	**18%**	[14%, 23%]	**34%**	[29%, 40%]	**48%**	[42%, 54%]	269
Hulu	**13%**	[9%, 18%]	**32%**	[26%, 38%]	**55%**	[49%, 61%]	225
Cable company	**8%**	[5%, 12%]	**38%**	[31%, 44%]	**55%**	[48%, 61%]	212
Venmo	**17%**	[12%, 24%]	**26%**	[20%, 33%]	**56%**	[49%, 63%]	179
Apple Pay	**21%**	[15%, 28%]	**30%**	[23%, 37%]	**50%**	[42%, 57%]	155
Zelle	**17%**	[11%, 24%]	**34%**	[27%, 42%]	**49%**	[41%, 58%]	138
Wayfair	**4%**	[2%, 8%]	**33%**	[25%, 41%]	**64%**	[55%, 71%]	135
Intuit	**9%**	[5%, 17%]	**24%**	[17%, 34%]	**66%**	[56%, 75%]	95
Chime	**33%**	[24%, 44%]	**24%**	[16%, 35%]	**42%**	[32%, 53%]	78
Coinbase	**19%**	[11%, 31%]	**38%**	[26%, 51%]	**43%**	[31%, 57%]	53
Afterpay	**22%**	[12%, 36%]	**20%**	[11%, 33%]	**59%**	[44%, 72%]	46
Checkr	**14%**	[7%, 27%]	**33%**	[20%, 47%]	**53%**	[39%, 67%]	43
Klarna	**25%**	[14%, 40%]	**20%**	[10%, 35%]	**55%**	[40%, 69%]	40
Tinder	**36%**	[20%, 55%]	**24%**	[11%, 43%]	**40%**	[23%, 59%]	25

Results show that a majority of consumers do not recall having been given a choice to opt out of mandatory arbitration—either because they cannot remember or because they think the opportunity to choose was not presented. (In some cases, they were correct. For instance, Hulu does not contain an opt-out provision in its standard terms [36]).

If respondents answered “Yes” to the above question, they were asked to elaborate on what steps users would need to take to opt out (e.g., “Below, please describe what, if any, steps [Venmo] users must take if they wish to opt out of the standard arbitration language”).

**Fig 2:**
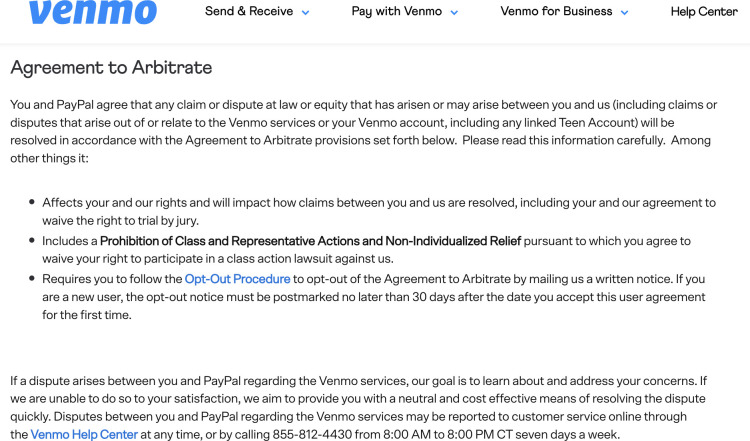
Venmo’s opt-out procedure.

Results indicate that few respondents retain information about how to opt out. For instance, none mentioned the limited time periods during which companies will accept opt outs. Several respondents mentioned steps that were either insufficient (e.g., “contact customer service”) or were too vague to demonstrate knowledge of the specific steps needed to opt out (e.g., “file the correct paperwork; “select opt out”). Notably, Venmo’s opt-out provision is featured prominently within its arbitration clause—the phrase “Opt Out Procedure” is a hyperlink printed in bright blue text (*See* Fig 2)—but even so, only 17% of Venmo users remembered they had been given the opportunity to opt out, and none could recall the steps that would need to be taken (Table 6.3).

Overall, the results provide little confidence that consumers take notice of or retain information about how to opt out of mandatory arbitration.

### Demographic variation in responses

The Appendix presents responses to the primary dependent variables separately by respondent gender, age, race/ethnicity, education, income, and legal experience. For the most part, systematic differences across sociodemographic groups were not observed. To the extent that significant sociodemographic predictors were observed, they were primarily legal background, education and, to a lesser extent, age and income.

## Conclusion

The purpose of this research was to update and extend Sovern and colleagues’ 2015 study assessing the quality of consent that consumers give to mandatory, predispute arbitration agreements, also known as “forced arbitration” agreements. The present research modernizes the prior study by examining how consumers interact with goods and services such as Venmo, Cash App, and Tinder.

The results show that most consumers do not focus on arbitration clauses when entering consumer contracts. Indeed, consumers spend little time reading contractual fine print, and they do not tend to factor in dispute resolution when deciding whether to use a product or service.

The present study also provides insight into the reasons why consumers’ decisions to sign up for products and services tend not to be affected by the presence of predispute arbitration clauses. When prompted, consumers report that they generally do not read the fine print, that they are unaware they are agreeing to submit all claims to binding arbitration, that they assume they have no meaningful choice about whether to agree to arbitration, and that they mistakenly believe that even after executing agreements containing mandatory arbitration provisions, they still retain the right to access the public courts.

The present findings underscore that consumers are generally unaware that they have agreed to mandatory arbitration in their own lives, and mistakenly assume that they retain many procedural rights that they, in fact, waive when they agree to arbitration. Indeed, nearly all respondents report holding an account with a company that requires binding arbitration as part of the standard terms and conditions, yet most respondents did not know whether they had (or erroneously asserted they had never) agreed to be bound by an arbitration clause. This finding raises questions about the quality of consent that consumers give to arbitration clauses.

Finally, this research shows that consumers are generally unaware of opt-out provisions, and hold mistaken beliefs about how one might effectuate an opt out if one prefers. Prior commentary has contended that one of the primary concerns about arbitration clauses is that “consumers do not fully understand the terms of these agreements, and that if they did, they cannot negotiate these terms, which are offered on a take-it-or-leave-it basis” [6]. This study highlights that even when the arbitration is offered *not* on a take-it-or-leave-it basis, but as a default option with an opt-out opportunity, consumers do not fully understand the opt-out terms, either. In summary, the present findings cast doubt on the notion that opt-out provisions enhance the quality of consent consumers provide when they submit to mandatory arbitration.

## Supporting information

S1 Appendix Appendix: Responses by demographic subgroups. (DOCX)
