# Supplementary material for: What do consumers understand about predispute arbitration agreements? an empirical investigation
Source: PLoS One. 2024 Feb 23;19(2):e0296179. doi: 10.1371/journal.pone.0296179 (PMC10889883; doi:10.1371/journal.pone.0296179)
Supplement: S1 Appendix — (DOCX) [file pone.0296179.s001.docx]

# Appendix

Table A1. Identity theft scenario– right to day in court

| **Would you have the right to have a court decide the dispute even if the bank did not want a court to decide the dispute?** | | | | |
| --- | --- | --- | --- | --- |
| **Demographic Covariates** | | **Yes** | **No** | **I don't know** |
| Full sample (n = 953) | | 0.56 (0.53, 0.59) | 0.16 (0.14, 0.18) | 0.28 (0.26, 0.31) |
| Gender | |  |  |  |
|  | Female (n = 500) | 0.59 (0.54, 0.63) | 0.11 (0.08, 0.14) | 0.31 (0.27, 0.35) |
|  | Male (n = 446) | 0.53 (0.48, 0.58) | 0.22 (0.18, 0.26) | 0.26 (0.22, 0.30) |
|  | Other gender identity (n = 7) | 0.29 (0.08, 0.64) | 0.14 (0.01, 0.51) | 0.57 (0.25, 0.84) |
| Age | |  |  |  |
|  | 18-34 years | 0.24 (0.20, 0.29) | 0.13 (0.10, 0.18) | 0.62 (0.57, 0.68) |
|  | 35-54 years | 0.28 (0.24, 0.33) | 0.14 (0.11, 0.18) | 0.58 (0.52, 0.63) |
|  | 55+ years | 0.32 (0.27, 0.37) | 0.19 (0.16, 0.24) | 0.49 (0.43, 0.54) |
| Race/Ethnicity | |  |  |  |
|  | White (including Middle Eastern or Arab) (n = 580) | 0.54 (0.50, 0.58) | 0.16 (0.13, 0.19) | 0.30 (0.26, 0.34) |
|  | Black/African-American (n = 114) | 0.70 (0.61, 0.78) | 0.11 (0.07, 0.19) | 0.18 (0.12, 0.27) |
|  | Hispanic/Latino/a (n = 155) | 0.55 (0.47, 0.62) | 0.16 (0.11, 0.23) | 0.29 (0.22, 0.37) |
|  | Asian (n = 58) | 0.53 (0.41, 0.66) | 0.17 (0.10, 0.29) | 0.29 (0.19, 0.42) |
|  | American Indian/Alaska Native (n = 8) | 0.62 (0.31, 0.86) | 0.25 (0.07, 0.59) | 0.12 (0.01, 0.47) |
|  | Native Hawaiian/Pacific Islander (n = 3) | 0.67 (0.21, 0.98) | 0.00 (0.00, 0.56) | 0.33 (0.02, 0.79) |
|  | Other (n = 10) | 0.40 (0.17, 0.69) | 0.20 (0.06, 0.51) | 0.40 (0.17, 0.69) |
|  | Chose one or more designation (n = 20) | 0.50 (0.30, 0.70) | 0.15 (0.05, 0.36) | 0.35 (0.18, 0.57) |
| Education | |  |  |  |
|  | Did not graduate from high school (n = 28) | 0.43 (0.27, 0.61) | 0.11 (0.04, 0.27) | 0.46 (0.30, 0.64) |
|  | High school graduate or GED (n = 217) | 0.63 (0.57, 0.69) | 0.14 (0.10, 0.20) | 0.23 (0.18, 0.29) |
|  | Some college or post-secondary work (n = 270) | 0.54 (0.48, 0.60) | 0.13 (0.10, 0.18) | 0.33 (0.28, 0.39) |
|  | College graduate (n = 291) | 0.57 (0.51, 0.63) | 0.15 (0.11, 0.20) | 0.28 (0.23, 0.33) |
|  | Post-graduate work (n= 147) | 0.48 (0.40, 0.56) | 0.25 (0.19, 0.33) | 0.27 (0.20, 0.34) |
| Income | |  |  |  |
|  | Less than $24,000 (n = 145) | 0.60 (0.52, 0.68) | 0.12 (0.08, 0.19) | 0.28 (0.21, 0.35) |
|  | At least $24,000 but less than $50,999 (n = 218) | 0.60 (0.53, 0.66) | 0.12 (0.09, 0.17) | 0.28 (0.22, 0.34) |
|  | At least $51,000 but less than $69,999 (n = 130) | 0.65 (0.56, 0.72) | 0.13 (0.08, 0.20) | 0.22 (0.16, 0.30) |
|  | At least $70,000 but less than $143,999 (n = 345) | 0.49 (0.44, 0.54) | 0.21 (0.17, 0.26) | 0.30 (0.25, 0.35) |
|  | At least $144,000 (n = 101) | 0.54 (0.45, 0.64) | 0.15 (0.09, 0.23) | 0.31 (0.23, 0.40) |
|  | Prefer not to answer (n = 13) | 0.31 (0.13, 0.58) | 0.08 (0.00, 0.33) | 0.62 (0.36, 0.82) |
| Legal Experience | |  |  |  |
|  | Lawyer or law student (n = 54) | 0.54 (0.51, 0.58) | 0.16 (0.14, 0.18) | 0.30 (0.27, 0.33) |
|  | Neither a lawyer nor law student (n = 899) | 0.80 (0.67, 0.88) | 0.17 (0.09, 0.29) | 0.04 (0.01, 0.13) |
| *Note*. 95% confidence intervals in parentheses. | |  |  |  |

Table A2. Identity theft scenario – right to jury trial

| **Under the terms of the contract you just saw, would you have a right to a jury trial?** | | | | |
| --- | --- | --- | --- | --- |
| **Demographic Covariates** | | **Yes** | **No** | **I don't know** |
| Full sample (n = 949) | | 0.48 (0.44, 0.51) | 0.22 (0.19, 0.24) | 0.31 (0.28, 0.34) |
| Gender | |  |  |  |
|  | Female (n = 496) | 0.51 (0.46, 0.55) | 0.17 (0.14, 0.20) | 0.33 (0.29, 0.37) |
|  | Male (n = 446) | 0.44 (0.40, 0.49) | 0.27 (0.23, 0.31) | 0.28 (0.24, 0.33) |
|  | Other gender identity (n = 7) | 0.29 (0.08, 0.64) | 0.14 (0.01, 0.51) | 0.57 (0.25, 0.84) |
| Age | |  |  |  |
|  | 18-34 years (n = 288) | 0.62 (0.56, 0.67) | 0.16 (0.12, 0.20) | 0.23 (0.18, 0.28) |
|  | 35-54 years (n = 317) | 0.47 (0.42, 0.53) | 0.22 (0.18, 0.27) | 0.30 (0.25, 0.36) |
|  | 55+ years (n = 342) | 0.36 (0.31, 0.41) | 0.26 (0.22, 0.31) | 0.38 (0.33, 0.43) |
| Race/Ethnicity | |  |  |  |
|  | White (including Middle Eastern or Arab) (n = 578) | 0.43 (0.39, 0.47) | 0.23 (0.20, 0.26) | 0.34 (0.30, 0.38) |
|  | Black/African-American (n = 114) | 0.61 (0.51, 0.69) | 0.18 (0.12, 0.27) | 0.21 (0.15, 0.29) |
|  | Hispanic/Latino/a (n = 153) | 0.56 (0.48, 0.64) | 0.19 (0.14, 0.26) | 0.25 (0.19, 0.32) |
|  | Asian (n = 58) | 0.43 (0.31, 0.56) | 0.22 (0.14, 0.35) | 0.34 (0.24, 0.47) |
|  | American Indian/Alaska Native (n = 8) | 0.50 (0.22, 0.78) | 0.25 (0.07, 0.59) | 0.25 (0.07, 0.59) |
|  | Native Hawaiian/Pacific Islander (n = 3) | 0.67 (0.21, 0.98) | 0.00 (0.00, 0.56) | 0.33 (0.02, 0.79) |
|  | Other (n = 10) | 0.40 (0.17, 0.69) | 0.40 (0.17, 0.69) | 0.20 (0.06, 0.51) |
|  | Chose one or more designation (n = 20) | 0.55 (0.34, 0.74) | 0.15 (0.05, 0.36) | 0.30 (0.15, 0.52) |
| Education | |  |  |  |
|  | Did not graduate from high school (n = 28) | 0.61 (0.42, 0.76) | 0.07 (0.02, 0.23) | 0.32 (0.18, 0.51) |
|  | High school graduate or GED (n = 215) | 0.56 (0.49, 0.62) | 0.21 (0.16, 0.27) | 0.23 (0.18, 0.29) |
|  | Some college or post-secondary work (n = 268) | 0.48 (0.42, 0.54) | 0.18 (0.14, 0.23) | 0.34 (0.28, 0.39) |
|  | College graduate (n = 291) | 0.47 (0.42, 0.53) | 0.20 (0.16, 0.25) | 0.33 (0.28, 0.38) |
|  | Post-graduate work (n= 147) | 0.32 (0.25, 0.40) | 0.35 (0.27, 0.43) | 0.33 (0.26, 0.41) |
| Income | |  |  |  |
|  | Less than $24,000 (n = 144) | 0.49 (0.41, 0.57) | 0.22 (0.16, 0.30) | 0.29 (0.22, 0.37) |
|  | At least $24,000 but less than $50,999 (n = 216) | 0.60 (0.53, 0.66) | 0.16 (0.11, 0.21) | 0.25 (0.19, 0.31) |
|  | At least $51,000 but less than $69,999 (n = 130) | 0.53 (0.45, 0.61) | 0.17 (0.11, 0.24) | 0.30 (0.23, 0.38) |
|  | At least $70,000 but less than $143,999 (n = 344) | 0.38 (0.33, 0.44) | 0.26 (0.22, 0.31) | 0.36 (0.31, 0.41) |
|  | At least $144,000 (n = 101) | 0.44 (0.34, 0.53) | 0.26 (0.18, 0.35) | 0.31 (0.23, 0.40) |
|  | Prefer not to answer (n = 13) | 0.46 (0.23, 0.71) | 0.15 (0.04, 0.42) | 0.38 (0.18, 0.64) |
| Legal Experience | |  |  |  |
|  | Lawyer or law student (n = 54) | 0.67 (0.53, 0.78) | 0.24 (0.15, 0.37) | 0.09 (0.04, 0.20) |
|  | Neither a lawyer nor law student (n = 895) | 0.46 (0.43, 0.50) | 0.21 (0.19, 0.24) | 0.32 (0.29, 0.35) |
| *Note*. 95% confidence intervals in parentheses. | |  |  |  |

Table A3. Identity theft scenario – right to participate in a class action

| **Could you be included with the other customers in a single lawsuit (that is, a class action lawsuit) against the bank?** | | | | |
| --- | --- | --- | --- | --- |
| **Demographic Covariates** | | **Yes** | **No** | **I don't know** |
| Full sample (n = 949) | | 0.60 (0.57, 0.63) | 0.14 (0.12, 0.17) | 0.25 (0.23, 0.28) |
| Gender | |  |  |  |
|  | Female (n = 496) | 0.60 (0.55, 0.64) | 0.13 (0.10, 0.16) | 0.28 (0.24, 0.32) |
|  | Male (n = 446) | 0.61 (0.57, 0.66) | 0.16 (0.13, 0.20) | 0.22 (0.19, 0.26) |
|  | Other gender identity (n = 7) | 0.43 (0.16, 0.75) | 0.14 (0.01, 0.51) | 0.43 (0.16, 0.75) |
| Age | |  |  |  |
|  | 18-34 years (n = 288) | 0.61 (0.55, 0.66) | 0.18 (0.14, 0.23) | 0.21 (0.17, 0.26) |
|  | 35-54 years (n = 317) | 0.62 (0.57, 0.67) | 0.14 (0.11, 0.18) | 0.24 (0.19, 0.29) |
|  | 55+ years (n = 343) | 0.59 (0.53, 0.64) | 0.12 (0.09, 0.15) | 0.30 (0.25, 0.35) |
| Race/Ethnicity | |  |  |  |
|  | White (including Middle Eastern or Arab) (n = 578) | 0.60 (0.55, 0.63) | 0.13 (0.10, 0.16) | 0.28 (0.24, 0.31) |
|  | Black/African-American (n = 114) | 0.63 (0.54, 0.71) | 0.19 (0.13, 0.27) | 0.18 (0.12, 0.26) |
|  | Hispanic/Latino/a (n = 153) | 0.61 (0.53, 0.68) | 0.15 (0.10, 0.22) | 0.24 (0.18, 0.32) |
|  | Asian (n = 58) | 0.53 (0.41, 0.66) | 0.21 (0.12, 0.33) | 0.26 (0.16, 0.38) |
|  | American Indian/Alaska Native (n = 8) | 0.88 (0.53, 0.99) | 0.12 (0.01, 0.47) | 0.00 (0.00, 0.32) |
|  | Native Hawaiian/Pacific Islander (n = 3) | 0.67 (0.21, 0.98) | 0.00 (0.00, 0.56) | 0.33 (0.02, 0.79) |
|  | Other (n = 10) | 0.80 (0.49, 0.94) | 0.10 (0.01, 0.40) | 0.10 (0.01, 0.40) |
|  | Chose one or more designation (n = 20) | 0.80 (0.58, 0.92) | 0.15 (0.05, 0.36) | 0.05 (0.00, 0.24) |
| Education | |  |  |  |
|  | Did not graduate from high school (n = 28) | 0.46 (0.30, 0.64) | 0.21 (0.10, 0.40) | 0.32 (0.18, 0.51) |
|  | High school graduate or GED (n = 215) | 0.58 (0.51, 0.64) | 0.20 (0.16, 0.26) | 0.22 (0.17, 0.28) |
|  | Some college or post-secondary work (n = 268) | 0.60 (0.54, 0.66) | 0.14 (0.11, 0.19) | 0.25 (0.21, 0.31) |
|  | College graduate (n = 291) | 0.64 (0.59, 0.70) | 0.11 (0.08, 0.15) | 0.24 (0.20, 0.30) |
|  | Post-graduate work (n= 147) | 0.59 (0.51, 0.67) | 0.11 (0.07, 0.17) | 0.30 (0.23, 0.38) |
| Income | |  |  |  |
|  | Less than $24,000 (n = 144) | 0.57 (0.49, 0.65) | 0.13 (0.09, 0.20) | 0.30 (0.23, 0.38) |
|  | At least $24,000 but less than $50,999 (n = 216) | 0.63 (0.56, 0.69) | 0.18 (0.13, 0.23) | 0.19 (0.15, 0.25) |
|  | At least $51,000 but less than $69,999 (n = 130) | 0.64 (0.55, 0.72) | 0.15 (0.10, 0.23) | 0.21 (0.15, 0.29) |
|  | At least $70,000 but less than $143,999 (n = 344) | 0.59 (0.53, 0.64) | 0.15 (0.11, 0.19) | 0.27 (0.22, 0.32) |
|  | At least $144,000 (n = 101) | 0.63 (0.54, 0.72) | 0.09 (0.05, 0.16) | 0.28 (0.20, 0.37) |
|  | Prefer not to answer (n = 13) | 0.38 (0.18, 0.64) | 0.08 (0.00, 0.33) | 0.54 (0.29, 0.77) |
| Legal Experience | |  |  |  |
|  | Lawyer or law student (n = 54) | 0.67 (0.53, 0.78) | 0.24 (0.15, 0.37) | 0.09 (0.04, 0.20) |
|  | Neither a lawyer nor law student (n = 895) | 0.60 (0.57, 0.63) | 0.14 (0.12, 0.16) | 0.26 (0.23, 0.29) |
| *Note*. 95% confidence intervals in parentheses. | |  |  |  |

Table A4. Identity theft scenario – right to appeal to another arbitrator

| **I could appeal the arbitrator's erroneous decision to another arbitrator or set of arbitrators.** | | | | |
| --- | --- | --- | --- | --- |
| **Demographic Covariates** | | **Agree** | **Disagree** | **Neither** |
| Full sample (n = 946) | | 0.62 (0.58, 0.65) | 0.07 (0.06, 0.09) | 0.31 (0.28, 0.34) |
| Gender | |  |  |  |
|  | Female (n = 495) | 0.59 (0.54, 0.63) | 0.06 (0.04, 0.09) | 0.35 (0.31, 0.39) |
|  | Male (n = 444) | 0.65 (0.60, 0.69) | 0.09 (0.07, 0.12) | 0.26 (0.22, 0.30) |
|  | Other gender identity (n = 7) | 0.57 (0.25, 0.84) | 0.00 (0.00, 0.35) | 0.43 (0.16, 0.75) |
| Age | |  |  |  |
|  | 18-34 years (n = 287) | 0.60 (0.55, 0.66) | 0.05 (0.03, 0.08) | 0.35 (0.30, 0.41) |
|  | 35-54 years (n = 316) | 0.64 (0.59, 0.69) | 0.07 (0.04, 0.10) | 0.29 (0.24, 0.34) |
|  | 55+ years (n = 342) | 0.61 (0.55, 0.66) | 0.11 (0.08, 0.14) | 0.29 (0.24, 0.34) |
| Race/Ethnicity | |  |  |  |
|  | White (including Middle Eastern or Arab) (n = 575) | 0.62 (0.58, 0.66) | 0.08 (0.06, 0.11) | 0.29 (0.26, 0.33) |
|  | Black/African-American (n = 114) | 0.59 (0.50, 0.67) | 0.04 (0.01, 0.09) | 0.38 (0.29, 0.47) |
|  | Hispanic/Latino/a (n = 153) | 0.63 (0.55, 0.70) | 0.06 (0.03, 0.11) | 0.31 (0.25, 0.39) |
|  | Asian (n = 58) | 0.66 (0.53, 0.76) | 0.07 (0.03, 0.16) | 0.28 (0.18, 0.40) |
|  | American Indian/Alaska Native (n = 8) | 0.62 (0.31, 0.86) | 0.25 (0.07, 0.59) | 0.12 (0.01, 0.47) |
|  | Native Hawaiian/Pacific Islander (n = 3) | 0.75 (0.41, 0.93) | 0.12 (0.01, 0.47) | 0.12 (0.01, 0.47) |
|  | Other (n = 10) | 0.30 (0.11, 0.60) | 0.20 (0.06, 0.51) | 0.50 (0.24, 0.76) |
|  | Chose one or more designation (n = 20) | 0.55 (0.34, 0.74) | 0.05 (0.00, 0.24) | 0.40 (0.22, 0.61) |
| Education | |  |  |  |
|  | Did not graduate from high school (n = 28) | 0.36 (0.21, 0.54) | 0.04 (0.00, 0.18) | 0.61 (0.42, 0.76) |
|  | High school graduate or GED (n = 214) | 0.57 (0.50, 0.63) | 0.04 (0.02, 0.07) | 0.40 (0.33, 0.46) |
|  | Some college or post-secondary work (n = 267) | 0.61 (0.55, 0.67) | 0.07 (0.04, 0.10) | 0.32 (0.27, 0.38) |
|  | College graduate (n = 290) | 0.69 (0.63, 0.74) | 0.08 (0.06, 0.12) | 0.23 (0.19, 0.28) |
|  | Post-graduate work (n= 147) | 0.61 (0.53, 0.69) | 0.13 (0.08, 0.19) | 0.26 (0.19, 0.33) |
| Income | |  |  |  |
|  | Less than $24,000 (n = 143) | 0.50 (0.42, 0.58) | 0.02 (0.01, 0.06) | 0.48 (0.40, 0.56) |
|  | At least $24,000 but less than $50,999 (n = 215) | 0.6 (0.53, 0.66) | 0.06 (0.04, 0.10) | 0.34 (0.28, 0.41) |
|  | At least $51,000 but less than $69,999 (n = 129) | 0.67 (0.59, 0.75) | 0.07 (0.04, 0.13) | 0.26 (0.19, 0.34) |
|  | At least $70,000 but less than $143,999 (n = 344) | 0.64 (0.58, 0.69) | 0.10 (0.07, 0.14) | 0.26 (0.22, 0.31) |
|  | At least $144,000 (n = 101) | 0.69 (0.60, 0.77) | 0.09 (0.05, 0.16) | 0.22 (0.15, 0.31) |
|  | Prefer not to answer (n = 13) | 0.62 (0.36, 0.82) | 0.00 (0.00, 0.23) | 0.38 (0.18, 0.64) |
| Legal Experience | |  |  |  |
|  | Lawyer or law student (n = 54) | 0.67 (0.53, 0.78) | 0.04 (0.01, 0.13) | 0.30 (0.19, 0.43) |
|  | Neither a lawyer nor law student (n = 892) | 0.61 (0.58, 0.64) | 0.08 (0.06, 0.10) | 0.31 (0.28, 0.34) |
| *Note*. 95% confidence intervals in parentheses. | |  |  |  |

Table A5. Identity theft scenario – right to start over again in court

| **I could set aside the arbitrator's erroneous decision and start all over again in court.** | | | | |
| --- | --- | --- | --- | --- |
| **Demographic Covariates** | | **Agree** | **Disagree** | **Neither** |
| Full sample (n = 946) | | 0.35 (0.32, 0.38) | 0.21 (0.19, 0.24) | 0.44 (0.41, 0.48) |
| Gender | |  |  |  |
|  | Female (n = 495) | 0.33 (0.29, 0.38) | 0.20 (0.16, 0.23) | 0.47 (0.43, 0.51) |
|  | Male (n = 444) | 0.36 (0.32, 0.41) | 0.23 (0.19, 0.27) | 0.41 (0.37, 0.46) |
|  | Other gender identity (n = 7) | 0.14 (0.01, 0.51) | 0.29 (0.08, 0.64) | 0.57 (0.25, 0.84) |
| Age | |  |  |  |
|  | 18-34 years (n = 287) | 0.39 (0.34, 0.45) | 0.16 (0.13, 0.21) | 0.45 (0.39, 0.50) |
|  | 35-54 years (n = 316) | 0.39 (0.33, 0.44) | 0.18 (0.14, 0.23) | 0.43 (0.38, 0.49) |
|  | 55+ years (n = 342) | 0.27 (0.23, 0.32) | 0.27 (0.23, 0.32) | 0.45 (0.40, 0.51) |
| Race/Ethnicity | |  |  |  |
|  | White (including Middle Eastern or Arab) (n = 575) | 0.32 (0.28, 0.36) | 0.24 (0.20, 0.27) | 0.44 (0.40, 0.48) |
|  | Black/African-American (n = 114) | 0.44 (0.35, 0.53) | 0.14 (0.09, 0.22) | 0.42 (0.33, 0.51) |
|  | Hispanic/Latino/a (n = 153) | 0.38 (0.31, 0.46) | 0.18 (0.13, 0.25) | 0.44 (0.36, 0.52) |
|  | Asian (n = 58) | 0.34 (0.24, 0.47) | 0.16 (0.08, 0.27) | 0.50 (0.38, 0.62) |
|  | American Indian/Alaska Native (n = 8) | 0.38 (0.14, 0.69) | 0.25 (0.07, 0.59) | 0.38 (0.14, 0.69) |
|  | Native Hawaiian/Pacific Islander (n = 3) | 0.33 (0.02, 0.79) | 0.00 (0.00, 0.56) | 0.67 (0.21, 0.98) |
|  | Other (n = 10) | 0.30 (0.11, 0.60) | 0.20 (0.06, 0.51) | 0.50 (0.24, 0.76) |
|  | Chose one or more designation (n = 20) | 0.35 (0.18, 0.57) | 0.25 (0.11, 0.47) | 0.40 (0.22, 0.61) |
| Education | |  |  |  |
|  | Did not graduate from high school (n = 28) | 0.36 (0.21, 0.54) | 0.04 (0.00, 0.18) | 0.61 (0.42, 0.76) |
|  | High school graduate or GED (n = 214) | 0.38 (0.32, 0.45) | 0.16 (0.12, 0.21) | 0.46 (0.39, 0.52) |
|  | Some college or post-secondary work (n = 267) | 0.36 (0.30, 0.42) | 0.20 (0.16, 0.25) | 0.44 (0.38, 0.50) |
|  | College graduate (n = 290) | 0.34 (0.29, 0.40) | 0.23 (0.18, 0.28) | 0.43 (0.38, 0.49) |
|  | Post-graduate work (n= 147) | 0.27 (0.21, 0.35) | 0.30 (0.23, 0.38) | 0.43 (0.35, 0.51) |
| Income | |  |  |  |
|  | Less than $24,000 (n = 143) | 0.34 (0.27, 0.42) | 0.14 (0.09, 0.21) | 0.52 (0.44, 0.60) |
|  | At least $24,000 but less than $50,999 (n = 215) | 0.33 (0.27, 0.40) | 0.19 (0.14, 0.24) | 0.48 (0.42, 0.55) |
|  | At least $51,000 but less than $69,999 (n = 129) | 0.44 (0.36, 0.53) | 0.18 (0.12, 0.25) | 0.38 (0.30, 0.47) |
|  | At least $70,000 but less than $143,999 (n = 344) | 0.33 (0.28, 0.38) | 0.25 (0.21, 0.30) | 0.42 (0.37, 0.47) |
|  | At least $144,000 (n = 101) | 0.33 (0.24, 0.42) | 0.27 (0.19, 0.36) | 0.41 (0.32, 0.50) |
|  | Prefer not to answer (n = 13) | 0.23 (0.08, 0.50) | 0.15 (0.04, 0.42) | 0.62 (0.36, 0.82) |
| Legal Experience | |  |  |  |
|  | Lawyer or law student (n = 54) | 0.54 (0.41, 0.66) | 0.06 (0.02, 0.15) | 0.41 (0.29, 0.54) |
|  | Neither a lawyer nor law student (n = 892) | 0.33 (0.30, 0.37) | 0.22 (0.19, 0.25) | 0.45 (0.41, 0.48) |
| *Note*. 95% confidence intervals in parentheses. | |  |  |  |

Table A6. Illegal detention scenario – right to day in court

| **Would you have a right to sue the bank in court?** | | | | |
| --- | --- | --- | --- | --- |
| **Demographic Covariates** | | **No** | **Yes** | **I don't know** |
| Full sample (n = 945) | | 0.20 (0.18, 0.23) | 0.58 (0.55, 0.61) | 0.22 (0.20, 0.25) |
| Gender | |  |  |  |
|  | Female (n = 494) | 0.20 (0.17, 0.24) | 0.55 (0.50, 0.59) | 0.25 (0.21, 0.29) |
|  | Male (n = 444) | 0.02 (0.17, 0.24) | 0.61 (0.56, 0.65) | 0.19 (0.16, 0.23) |
|  | Other gender identity (n = 7) | 0.14 (0.01, 0.51) | 0.71 (0.36, 0.92) | 0.14 (0.01, 0.51) |
| Age | |  |  |  |
|  | 18-34 years (n = 287) | 0.22 (0.17, 0.27) | 0.64 (0.58, 0.69) | 0.14 (0.11, 0.19) |
|  | 35-54 years (n = 315) | 0.24 (0.20, 0.29) | 0.55 (0.49, 0.60) | 0.21 (0.17, 0.26) |
|  | 55+ years (n = 342) | 0.15 (0.12, 0.19) | 0.55 (0.50, 0.60) | 0.30 (0.25, 0.35) |
| Race/Ethnicity | |  |  |  |
|  | White (including Middle Eastern or Arab) (n = 574) | 0.19 (0.16, 0.22) | 0.58 (0.54, 0.62) | 0.23 (0.20, 0.27) |
|  | Black/African-American (n = 114) | 0.30 (0.22, 0.39) | 0.57 (0.48, 0.66) | 0.13 (0.08, 0.21) |
|  | Hispanic/Latino/a (n = 153) | 0.18 (0.12, 0.24) | 0.58 (0.50, 0.66) | 0.24 (0.18, 0.32) |
|  | Asian (n = 58) | 0.21 (0.12, 0.33) | 0.55 (0.42, 0.67) | 0.24 (0.15, 0.37) |
|  | American Indian/Alaska Native (n = 8) | 0.25 (0.07, 0.59) | 0.62 (0.31, 0.86) | 0.12 (0.01, 0.47) |
|  | Native Hawaiian/Pacific Islander (n = 3) | 0.00 (0.00, 0.56) | 0.67 (0.21, 0.98) | 0.33 (0.02, 0.79) |
|  | Other (n = 10) | 0.30 (0.11, 0.60) | 0.50 (0.24, 0.76) | 0.20 (0.06, 0.51) |
|  | Chose one or more designation (n = 20) | 0.15 (0.05, 0.36) | 0.70 (0.48, 0.85) | 0.15 (0.05, 0.36) |
| Education | |  |  |  |
|  | Did not graduate from high school (n = 28) | 0.07 (0.02, 0.23) | 0.61 (0.42, 0.76) | 0.32 (0.18, 0.51) |
|  | High school graduate or GED (n = 213) | 0.23 (0.18, 0.30) | 0.58 (0.51, 0.64) | 0.19 (0.14, 0.25) |
|  | Some college or post-secondary work (n = 267) | 0.19 (0.15, 0.24) | 0.57 (0.51, 0.62) | 0.24 (0.20, 0.30) |
|  | College graduate (n = 290) | 0.19 (0.15, 0.24) | 0.59 (0.54, 0.65) | 0.22 (0.17, 0.27) |
|  | Post-graduate work (n= 147) | 0.22 (0.16, 0.29) | 0.56 (0.48, 0.64) | 0.22 (0.16, 0.29) |
| Income | |  |  |  |
|  | Less than $24,000 (n = 143) | 0.20 (0.14, 0.27) | 0.60 (0.52, 0.68) | 0.20 (0.15, 0.28) |
|  | At least $24,000 but less than $50,999 (n = 215) | 0.20 (0.16, 0.26) | 0.55 (0.48, 0.61) | 0.25 (0.19, 0.31) |
|  | At least $51,000 but less than $69,999 (n = 129) | 0.22 (0.16, 0.30) | 0.57 (0.48, 0.65) | 0.21 (0.15, 0.29) |
|  | At least $70,000 but less than $143,999 (n = 343) | 0.20 (0.16, 0.25) | 0.57 (0.52, 0.62) | 0.23 (0.19, 0.27) |
|  | At least $144,000 (n = 101) | 0.20 (0.13, 0.29) | 0.62 (0.53, 0.71) | 0.18 (0.12, 0.26) |
|  | Prefer not to answer (n = 13) | 0.00 (0.00, 0.23) | 0.69 (0.42, 0.87) | 0.31 (0.13, 0.58) |
| Legal Experience | |  |  |  |
|  | Lawyer or law student (n = 54) | 0.20 (0.12, 0.33) | 0.76 (0.63, 0.85) | 0.04 (0.01, 0.13) |
|  | Neither a lawyer nor law student (n = 891) | 0.20 (0.18, 0.23) | 0.57 (0.53, 0.60) | 0.23 (0.21, 0.26) |
| *Note*. 95% confidence intervals in parentheses. | |  |  |  |

Table A7. Illegal detention scenario – right to participate in a class action

| **Could you be included with the other customers in a single lawsuit (that is, a class action lawsuit) against the bank?** | | | | |
| --- | --- | --- | --- | --- |
| **Demographic Covariates** | | **No** | **Yes** | **I don't know** |
| Full sample (n = 945) | | 0.18 (0.16, 0.21) | 0.56 (0.53, 0.59) | 0.26 (0.23, 0.28) |
| Gender | |  |  |  |
|  | Female (n = 494) | 0.17 (0.14, 0.20) | 0.54 (0.50, 0.59) | 0.29 (0.25, 0.33) |
|  | Male (n = 444) | 0.20 (0.17, 0.24) | 0.58 (0.53, 0.62) | 0.22 (0.18, 0.26) |
|  | Other gender identity (n = 7) | 0.14 (0.01, 0.51) | 0.71 (0.36, 0.92) | 0.14 (0.01, 0.51) |
| Age | |  |  |  |
|  | 18-34 years (n = 287) | 0.18 (0.14, 0.23) | 0.63 (0.57, 0.68) | 0.19 (0.15, 0.24) |
|  | 35-54 years (n = 315) | 0.21 (0.17, 0.25) | 0.55 (0.50, 0.61) | 0.24 (0.20, 0.29) |
|  | 55+ years (n = 342) | 0.17 (0.13, 0.21) | 0.51 (0.46, 0.56) | 0.32 (0.27, 0.37) |
| Race/Ethnicity | |  |  |  |
|  | White (including Middle Eastern or Arab) (n = 574) | 0.20 (0.17, 0.23) | 0.53 (0.49, 0.58) | 0.27 (0.23, 0.31) |
|  | Black/African-American (n = 114) | 0.20 (0.14, 0.28) | 0.60 (0.50, 0.68) | 0.20 (0.14, 0.28) |
|  | Hispanic/Latino/a (n = 153) | 0.13 (0.09, 0.19) | 0.62 (0.54, 0.69) | 0.25 (0.19, 0.32) |
|  | Asian (n = 58) | 0.12 (0.06, 0.23) | 0.62 (0.49, 0.73) | 0.26 (0.16, 0.38) |
|  | American Indian/Alaska Native (n = 8) | 0.50 (0.22, 0.78) | 0.38 (0.14, 0.69) | 0.12 (0.01, 0.47) |
|  | Native Hawaiian/Pacific Islander (n = 3) | 0.00 (0.00, 0.56) | 0.67 (0.21, 0.98) | 0.33 (0.02, 0.79) |
|  | Other (n = 10) | 0.20 (0.06, 0.51) | 0.50 (0.24, 0.76) | 0.30 (0.11, 0.60) |
|  | Chose one or more designation (n = 20) | 0.20 (0.08, 0.42) | 0.65 (0.43, 0.82) | 0.15 (0.05, 0.36) |
| Education | |  |  |  |
|  | Did not graduate from high school (n = 28) | 0.11 (0.04, 0.27) | 0.50 (0.33, 0.67) | 0.39 (0.24, 0.58) |
|  | High school graduate or GED (n = 213) | 0.22 (0.17, 0.28) | 0.57 (0.50, 0.63) | 0.22 (0.17, 0.28) |
|  | Some college or post-secondary work (n = 267) | 0.18 (0.14, 0.23) | 0.57 (0.51, 0.62) | 0.25 (0.20, 0.31) |
|  | College graduate (n = 290) | 0.16 (0.12, 0.21) | 0.60 (0.54, 0.65) | 0.24 (0.20, 0.29) |
|  | Post-graduate work (n= 147) | 0.20 (0.15, 0.28) | 0.48 (0.40, 0.56) | 0.32 (0.25, 0.40) |
| Income | |  |  |  |
|  | Less than $24,000 (n = 143) | 0.22 (0.16, 0.30) | 0.52 (0.44, 0.60) | 0.25 (0.19, 0.33) |
|  | At least $24,000 but less than $50,999 (n = 215) | 0.15 (0.11, 0.20) | 0.59 (0.52, 0.65) | 0.27 (0.21, 0.33) |
|  | At least $51,000 but less than $69,999 (n = 129) | 0.21 (0.15, 0.29) | 0.60 (0.52, 0.68) | 0.19 (0.13, 0.26) |
|  | At least $70,000 but less than $143,999 (n = 343) | 0.20 (0.16, 0.24) | 0.53 (0.47, 0.58) | 0.27 (0.23, 0.32) |
|  | At least $144,000 (n = 101) | 0.15 (0.09, 0.23) | 0.60 (0.51, 0.69) | 0.25 (0.17, 0.34) |
|  | Prefer not to answer (n = 13) | 0.00 (0.00, 0.23) | 0.62 (0.36, 0.82) | 0.38 (0.18, 0.64) |
| Legal Experience | |  |  |  |
|  | Lawyer or law student (n = 54) | 0.24 (0.15, 0.37) | 0.67 (0.53, 0.78) | 0.09 (0.04, 0.20) |
|  | Neither a lawyer nor law student (n = 891) | 0.18 (0.16, 0.21) | 0.55 (0.52, 0.59) | 0.26 (0.24, 0.29) |
| *Note*. 95% confidence intervals in parentheses. | |  |  |  |

Table A8. How much experience do respondents have with arbitration clauses?

| **Have you ever entered into a consumer contract with a company that said you must arbitrate any disputes (and therefore cannot sue the company)?** | | | | |
| --- | --- | --- | --- | --- |
| **Demographic Covariates** | | **Yes** | **No** | **Unsure** |
| Full sample (n = 945) | | 0.17 (0.14, 0.19) | 0.48 (0.45, 0.51) | 0.35 (0.32, 0.38) |
| Gender | |  |  |  |
|  | Female (n = 494) | 0.13 (0.11, 0.17) | 0.53 (0.48, 0.57) | 0.34 (0.30, 0.38) |
|  | Male (n = 444) | 0.20 (0.17, 0.24) | 0.43 (0.39, 0.48) | 0.36 (0.32, 0.41) |
|  | Other gender identity (n = 7) | 0.00 (0.00, 0.35) | 0.29 (0.08, 0.64) | 0.71 (0.36, 0.92) |
| Age | |  |  |  |
|  | 18-34 years (n = 287) | 0.14 (0.10, 0.18) | 0.53 (0.48, 0.59) | 0.33 (0.28, 0.39) |
|  | 35-54 years (n = 315) | 0.15 (0.11, 0.19) | 0.48 (0.43, 0.54) | 0.37 (0.32, 0.42) |
|  | 55+ years (n = 342) | 0.21 (0.17, 0.25) | 0.44 (0.38, 0.49) | 0.36 (0.31, 0.41) |
| Race/Ethnicity | |  |  |  |
|  | White (including Middle Eastern or Arab) (n = 574) | 0.18 (0.15, 0.21) | 0.45 (0.41, 0.50) | 0.37 (0.33, 0.41) |
|  | Black/African-American (n = 114) | 0.11 (0.07, 0.19) | 0.56 (0.47, 0.65) | 0.32 (0.25, 0.42) |
|  | Hispanic/Latino/a (n = 153) | 0.15 (0.10, 0.22) | 0.50 (0.42, 0.58) | 0.35 (0.28, 0.42) |
|  | Asian (n = 58) | 0.16 (0.08, 0.27) | 0.53 (0.41, 0.66) | 0.31 (0.21, 0.44) |
|  | American Indian/Alaska Native (n = 8) | 0.25 (0.07, 0.59) | 0.38 (0.14, 0.69) | 0.38 (0.14, 0.69) |
|  | Native Hawaiian/Pacific Islander (n = 3) | 0.00 (0.00, 0.56) | 0.33 (0.02, 0.79) | 0.67 (0.21, 0.98) |
|  | Other (n = 10) | 0.20 (0.06, 0.51) | 0.60 (0.31, 0.83) | 0.20 (0.06, 0.51) |
|  | Chose one or more designation (n = 20) | 0.25 (0.11, 0.47) | 0.55 (0.34, 0.74) | 0.20 (0.08, 0.42) |
| Education | |  |  |  |
|  | Did not graduate from high school (n = 28) | 0.07 (0.02, 0.23) | 0.64 (0.46, 0.79) | 0.29 (0.15, 0.47) |
|  | High school graduate or GED (n = 213) | 0.08 (0.05, 0.12) | 0.63 (0.57, 0.70) | 0.29 (0.23, 0.35) |
|  | Some college or post-secondary work (n = 267) | 0.15 (0.12, 0.20) | 0.43 (0.37, 0.49) | 0.42 (0.36, 0.48) |
|  | College graduate (n = 290) | 0.20 (0.15, 0.25) | 0.46 (0.40, 0.51) | 0.35 (0.30, 0.40) |
|  | Post-graduate work (n= 147) | 0.27 (0.21, 0.35) | 0.38 (0.31, 0.46) | 0.35 (0.27, 0.43) |
| Income | |  |  |  |
|  | Less than $24,000 (n = 143) | 0.10 (0.06, 0.17) | 0.60 (0.52, 0.68) | 0.29 (0.23, 0.37) |
|  | At least $24,000 but less than $50,999 (n = 215) | 0.11 (0.08, 0.16) | 0.54 (0.48, 0.61) | 0.34 (0.28, 0.41) |
|  | At least $51,000 but less than $69,999 (n = 129) | 0.09 (0.05, 0.16) | 0.54 (0.46, 0.63) | 0.36 (0.29, 0.45) |
|  | At least $70,000 but less than $143,999 (n = 343) | 0.22 (0.18, 0.26) | 0.41 (0.36, 0.46) | 0.38 (0.33, 0.43) |
|  | At least $144,000 (n = 101) | 0.31 (0.23, 0.40) | 0.35 (0.26, 0.44) | 0.35 (0.26, 0.44) |
|  | Prefer not to answer (n = 13) | 0.08 (0.00, 0.33) | 0.54 (0.29, 0.77) | 0.38 (0.18, 0.64) |
| Legal Experience | |  |  |  |
|  | Lawyer or law student (n = 54) | 0.24 (0.15, 0.37) | 0.48 (0.35, 0.61) | 0.28 (0.18, 0.41) |
|  | Neither a lawyer nor law student (n = 891) | 0.16 (0.14, 0.19) | 0.48 (0.45, 0.51) | 0.36 (0.33, 0.39) |
| *Note*. 95% confidence intervals in parentheses. | |  |  |  |

Table A9. Does arbitration affect consumers’ contracting decisions ex ante?

| **Have you ever decided to use a product or service based on whether the terms and conditions contain an arbitration clause?** | | | |
| --- | --- | --- | --- |
| **Demographic Covariates** | | **Yes** | **No** |
| Full sample (n = 945) | | 0.07 (0.06, 0.09) | 0.93 (0.91, 0.94) |
| Gender | |  |  |
|  | Female (n = 494) | 0.07 (0.05, 0.09) | 0.93 (0.91, 0.95) |
|  | Male (n = 444) | 0.08 (0.06, 0.11) | 0.92 (0.89, 0.94) |
|  | Other gender identity (n = 7) | 0.00 (0.00, 0.35) | 1.00 (0.65, 1.00) |
| Age | |  |  |
|  | 18-34 years (n = 287) | 0.07 (0.05, 0.11) | 0.93 (0.89, 0.95) |
|  | 35-54 years (n = 315) | 0.05 (0.03, 0.08) | 0.95 (0.92, 0.97) |
|  | 55+ years (n = 342) | 0.08 (0.06, 0.12) | 0.92 (0.88, 0.94) |
| Race/Ethnicity | |  |  |
|  | White (including Middle Eastern or Arab) (n = 574) | 0.07 (0.06, 0.10) | 0.93 (0.90, 0.94) |
|  | Black/African-American (n = 114) | 0.07 (0.04, 0.13) | 0.93 (0.87, 0.96) |
|  | Hispanic/Latino/a (n = 153) | 0.07 (0.04, 0.12) | 0.93 (0.88, 0.96) |
|  | Asian (n = 58) | 0.09 (0.04, 0.19) | 0.91 (0.81, 0.96) |
|  | American Indian/Alaska Native (n = 8) | 0.00 (0.00, 0.32) | 1.00 (0.68, 1.00) |
|  | Native Hawaiian/Pacific Islander (n = 3) | 0.00 (0.00, 0.56) | 1.00 (0.44, 1.00) |
|  | Other (n = 10) | 0.00 (0.00, 0.28) | 1.00 (0.72, 1.00) |
|  | Chose one or more designation (n = 20) | 0.00 (0.00, 0.16) | 1.00 (0.84, 1.00) |
| Education | |  |  |
|  | Did not graduate from high school (n = 28) | 0.07 (0.02, 0.23) | 0.93 (0.77, 0.98) |
|  | High school graduate or GED (n = 213) | 0.02 (0.01, 0.05) | 0.98 (0.95, 0.99) |
|  | Some college or post-secondary work (n = 267) | 0.07 (0.05, 0.11) | 0.93 (0.89, 0.95) |
|  | College graduate (n = 290) | 0.10 (0.07, 0.14) | 0.90 (0.86, 0.93) |
|  | Post-graduate work (n= 147) | 0.09 (0.05, 0.15) | 0.91 (0.85, 0.95) |
| Income | |  |  |
|  | Less than $24,000 (n = 143) | 0.04 (0.02, 0.09) | 0.96 (0.91, 0.98) |
|  | At least $24,000 but less than $50,999 (n = 215) | 0.05 (0.03, 0.08) | 0.95 (0.92, 0.97) |
|  | At least $51,000 but less than $69,999 (n = 129) | 0.03 (0.01, 0.08) | 0.97 (0.92, 0.99) |
|  | At least $70,000 but less than $143,999 (n = 343) | 0.10 (0.07, 0.13) | 0.90 (0.87, 0.93) |
|  | At least $144,000 (n = 101) | 0.13 (0.08, 0.21) | 0.87 (0.79, 0.92) |
|  | Prefer not to answer (n = 13) | 0.08 (0.00, 0.33) | 0.92 (0.67, 1.00) |
| Legal Experience | |  |  |
|  | Lawyer or law student (n = 54) | 0.13 (0.06, 0.24) | 0.87 (0.76, 0.94) |
|  | Neither a lawyer nor law student (n = 891) | 0.07 (0.05, 0.09) | 0.93 (0.91, 0.95) |
| *Note*. 95% confidence intervals in parentheses. | |  |  |

Table A10.

| **Are you allowed to create an account with the bank, WITHOUT accepting the part of the agreement that says all disputes will be resolved only in arbitration?** | | | | |
| --- | --- | --- | --- | --- |
| **Demographic Covariates** | | **Yes** | **No** | **I don't know** |
| Full sample (n = 944) | | 0.18 (0.15, 0.20) | 0.49 (0.46, 0.53) | 0.33 (0.30, 0.36) |
| Gender | |  |  |  |
|  | Female (n = 493) | 0.16 (0.13, 0.20) | 0.49 (0.45, 0.53) | 0.35 (0.31, 0.39) |
|  | Male (n = 444) | 0.19 (0.16, 0.23) | 0.50 (0.46, 0.55) | 0.30 (0.26, 0.35) |
|  | Other gender identity (n = 7) | 0.00 (0.00, 0.35) | 0.14 (0.01, 0.51) | 0.86 (0.49, 0.99) |
| Age | |  |  |  |
|  | 18-34 years (n = 287) | 0.21 (0.17, 0.26) | 0.54 (0.49, 0.60) | 0.24 (0.20, 0.30) |
|  | 35-54 years (n = 315) | 0.20 (0.16, 0.25) | 0.50 (0.44, 0.55) | 0.30 (0.25, 0.35) |
|  | 55+ years (n = 341) | 0.12 (0.09, 0.16) | 0.45 (0.40, 0.50) | 0.43 (0.37, 0.48) |
| Race/Ethnicity | |  |  |  |
|  | White (including Middle Eastern or Arab) (n = 573) | 0.16 (0.13, 0.19) | 0.47 (0.43, 0.52) | 0.36 (0.33, 0.40) |
|  | Black/African-American (n = 114) | 0.24 (0.17, 0.32) | 0.54 (0.45, 0.63) | 0.22 (0.15, 0.30) |
|  | Hispanic/Latino/a (n = 153) | 0.23 (0.17, 0.30) | 0.51 (0.43, 0.59) | 0.26 (0.20, 0.34) |
|  | Asian (n = 58) | 0.12 (0.06, 0.23) | 0.53 (0.41, 0.66) | 0.34 (0.24, 0.47) |
|  | American Indian/Alaska Native (n = 8) | 0.12 (0.01, 0.47) | 0.62 (0.31, 0.86) | 0.25 (0.07, 0.59) |
|  | Native Hawaiian/Pacific Islander (n = 3) | 0.33 (0.02, 0.79) | 0.33 (0.02, 0.79) | 0.33 (0.02, 0.79) |
|  | Other (n = 10) | 0.10 (0.01, 0.40) | 0.60 (0.31, 0.83) | 0.30 (0.11, 0.60) |
|  | Chose one or more designation (n = 20) | 0.10 (0.03, 0.30) | 0.50 (0.30, 0.70) | 0.40 (0.22, 0.61) |
| Education | |  |  |  |
|  | Did not graduate from high school (n = 28) | 0.14 (0.06, 0.31) | 0.46 (0.30, 0.64) | 0.39 (0.24, 0.58) |
|  | High school graduate or GED (n = 213) | 0.25 (0.20, 0.32) | 0.46 (0.39, 0.53) | 0.29 (0.23, 0.35) |
|  | Some college or post-secondary work (n = 266) | 0.12 (0.09, 0.17) | 0.50 (0.44, 0.56) | 0.38 (0.32, 0.44) |
|  | College graduate (n = 290) | 0.21 (0.16, 0.26) | 0.48 (0.43, 0.54) | 0.31 (0.26, 0.37) |
|  | Post-graduate work (n= 147) | 0.10 (0.06, 0.16) | 0.56 (0.48, 0.64) | 0.33 (0.26, 0.41) |
| Income | |  |  |  |
|  | Less than $24,000 (n = 143) | 0.15 (0.10, 0.22) | 0.46 (0.38, 0.54) | 0.38 (0.31, 0.47) |
|  | At least $24,000 but less than $50,999 (n = 215) | 0.18 (0.13, 0.23) | 0.54 (0.47, 0.60) | 0.28 (0.23, 0.35) |
|  | At least $51,000 but less than $69,999 (n = 128) | 0.24 (0.18, 0.32) | 0.49 (0.41, 0.58) | 0.27 (0.20, 0.35) |
|  | At least $70,000 but less than $143,999 (n = 343) | 0.16 (0.13, 0.21) | 0.48 (0.43, 0.54) | 0.35 (0.30, 0.40) |
|  | At least $144,000 (n = 101) | 0.17 (0.11, 0.25) | 0.51 (0.42, 0.61) | 0.32 (0.23, 0.41) |
|  | Prefer not to answer (n = 13) | 0.15 (0.04, 0.42) | 0.23 (0.08, 0.50) | 0.62 (0.36, 0.82) |
| Legal Experience | |  |  |  |
|  | Lawyer or law student (n = 54) | 0.44 (0.32, 0.58) | 0.48 (0.35, 0.61) | 0.07 (0.03, 0.18) |
|  | Neither a lawyer nor law student (n = 890) | 0.16 (0.14, 0.19) | 0.50 (0.46, 0.53) | 0.34 (0.31, 0.38) |
| *Note*. 95% confidence intervals in parentheses. | |  |  |  |

Table A11. Awareness of opt-out provisions

| **Did any of [Netflix/Hulu/Venmo/etc.] allow you to opt out of the portion of the terms and services that said that disputes would be resolved through arbitration?** | | | | |
| --- | --- | --- | --- | --- |
| **Demographic Covariates** | | **Yes** | **No** | **I don't know** |
| Full sample (n = 943) | | 0.21 (0.18, 0.24) | 0.30 (0.27, 0.33) | 0.49 (0.46, 0.52) |
| Gender | |  |  |  |
|  | Female (n = 493) | 0.21 (0.18, 0.25) | 0.28 (0.24, 0.32) | 0.51 (0.47, 0.56) |
|  | Male (n = 443) | 0.21 (0.17, 0.25) | 0.33 (0.29, 0.37) | 0.46 (0.42, 0.51) |
|  | Other gender identity (n = 7) | 0.00 (0.00, 0.35) | 0.29 (0.08, 0.64) | 0.71 (0.36, 0.92) |
| Age | |  |  |  |
|  | 18-34 years (n = 286) | 0.30 (0.25, 0.36) | 0.36 (0.30, 0.41) | 0.34 (0.29, 0.40) |
|  | 35-54 years (n = 315) | 0.23 (0.19, 0.28) | 0.30 (0.25, 0.35) | 0.47 (0.41, 0.52) |
|  | 55+ years (n = 341) | 0.11 (0.08, 0.15) | 0.26 (0.21, 0.30) | 0.64 (0.58, 0.69) |
| Race/Ethnicity | |  |  |  |
|  | White (including Middle Eastern or Arab) (n = 573) | 0.17 (0.14, 0.20) | 0.28 (0.24, 0.32) | 0.55 (0.51, 0.59) |
|  | Black/African-American (n = 114) | 0.30 (0.22, 0.39) | 0.43 (0.34, 0.52) | 0.27 (0.20, 0.36) |
|  | Hispanic/Latino/a (n = 152) | 0.28 (0.22, 0.36) | 0.28 (0.22, 0.36) | 0.43 (0.36, 0.51) |
|  | Asian (n = 58) | 0.22 (0.14, 0.35) | 0.31 (0.21, 0.44) | 0.47 (0.34, 0.59) |
|  | American Indian/Alaska Native (n = 8) | 0.00 (0.00, 0.32) | 0.38 (0.14, 0.69) | 0.62 (0.31, 0.86) |
|  | Native Hawaiian/Pacific Islander (n = 3) | 0.33 (0.02, 0.79) | 0.33 (0.02, 0.79) | 0.33 (0.02, 0.79) |
|  | Other (n = 10) | 0.20 (0.06, 0.51) | 0.50 (0.24, 0.76) | 0.30 (0.11, 0.06) |
|  | Chose one or more designation (n = 20) | 0.35 (0.18, 0.57) | 0.25 (0.11, 0.47) | 0.40 (0.22, 0.61) |
| Education | |  |  |  |
|  | Did not graduate from high school (n = 28) | 0.25 (0.13, 0.43) | 0.07 (0.02, 0.23) | 0.68 (0.49, 0.82) |
|  | High school graduate or GED (n = 213) | 0.29 (0.23, 0.35) | 0.31 (0.26, 0.38) | 0.40 (0.34, 0.47) |
|  | Some college or post-secondary work (n = 266) | 0.20 (0.16, 0.25) | 0.28 (0.23, 0.34) | 0.52 (0.46, 0.58) |
|  | College graduate (n = 289) | 0.19 (0.15, 0.24) | 0.33 (0.27, 0.38) | 0.48 (0.42, 0.54) |
|  | Post-graduate work (n= 147) | 0.14 (0.09, 0.20) | 0.31 (0.24, 0.39) | 0.55 (0.47, 0.63) |
| Income | |  |  |  |
|  | Less than $24,000 (n = 1432) | 0.20 (0.14, 0.27) | 0.30 (0.23, 0.38) | 0.50 (0.42, 0.58) |
|  | At least $24,000 but less than $50,999 (n = 215) | 0.26 (0.20, 0.32) | 0.32 (0.26, 0.39) | 0.42 (0.36, 0.49) |
|  | At least $51,000 but less than $69,999 (n = 128) | 0.22 (0.16, 0.30) | 0.24 (0.18, 0.32) | 0.54 (0.45, 0.62) |
|  | At least $70,000 but less than $143,999 (n = 343) | 0.19 (0.15, 0.23) | 0.30 (0.26, 0.35) | 0.51 (0.45, 0.56) |
|  | At least $144,000 (n = 101) | 0.17 (0.11, 0.25) | 0.36 (0.27, 0.45) | 0.48 (0.38, 0.57) |
|  | Prefer not to answer (n = 13) | 0.31 (0.13, 0.58) | 0.00 (0.00, 0.23) | 0.69 (0.42, 0.87) |
| Legal Experience | |  |  |  |
|  | Lawyer or law student (n = 54) | 0.46 (0.34, 0.59) | 0.37 (0.25, 0.50) | 0.17 (0.09, 0.29) |
|  | Neither a lawyer nor law student (n = 889) | 0.19 (0.17, 0.22) | 0.30 (0.27, 0.33) | 0.51 (0.48, 0.54) |
| *Note*. 95% confidence intervals in parentheses. | |  |  |  |
